# Deciding Not to Decide: Computational and Neural Evidence for Hidden Behavior in Sequential Choice

**DOI:** 10.1371/journal.pcbi.1003309

**Published:** 2013-10-31

**Authors:** Sebastian Gluth, Jörg Rieskamp, Christian Büchel

**Affiliations:** 1 Department of Systems Neuroscience, University Medical Center Hamburg-Eppendorf, Hamburg, Germany; 2 Department of Psychology, University of Basel, Basel, Switzerland; 3 Department of Psychology, Stanford University, Stanford, California, United States of America; University of Oxford, United Kingdom

## Abstract

Understanding the cognitive and neural processes that underlie human decision making requires the successful prediction of how, but also of when, people choose. Sequential sampling models (SSMs) have greatly advanced the decision sciences by assuming decisions to emerge from a bounded evidence accumulation process so that response times (RTs) become predictable. Here, we demonstrate a difficulty of SSMs that occurs when people are not forced to respond at once but are allowed to sample information sequentially: The decision maker might decide to delay the choice and terminate the accumulation process temporarily, a scenario not accounted for by the standard SSM approach. We developed several SSMs for predicting RTs from two independent samples of an electroencephalography (EEG) and a functional magnetic resonance imaging (fMRI) study. In these studies, participants bought or rejected fictitious stocks based on sequentially presented cues and were free to respond at any time. Standard SSM implementations did not describe RT distributions adequately. However, by adding a mechanism for postponing decisions to the model we obtained an accurate fit to the data. Time-frequency analysis of EEG data revealed alternating states of de- and increasing oscillatory power in beta-band frequencies (14–30 Hz), indicating that responses were repeatedly prepared and inhibited and thus lending further support for the existence of a decision not to decide. Finally, the extended model accounted for the results of an adapted version of our paradigm in which participants had to press a button for sampling more information. Our results show how computational modeling of decisions and RTs support a deeper understanding of the hidden dynamics in cognition.

## Author Summary

When decisions are made under uncertainty, we often decide not to choose immediately but to search for more information that reduces the uncertainty. In most psychological experiments, however, participants are forced to choose at once and cognitive models do not account for the possibility of deliberately delaying decisions. By modeling RT distributions in a sequential choice paradigm, we demonstrate that people decide not to decide when given the opportunity to sample more information. Importantly, this explicit decision to wait is distinguishable from an implicit delay in ongoing decisions as it actively inhibits this ongoing process. We then looked at EEG spectral power in the beta-band (frequencies from 14 to 30 Hz), which is known to reflect both the preparation and inhibition of responses. The obtained pattern is consistent with our proposal that participants repeatedly alternated between considering and postponing their decision in the sequential task. In an additional behavioral experiment, we show that our model also predicts RTs of the decision to sample more information. Hence, our combination of cognitive modeling and EEG provides converging evidence for the existence of a decision that is usually not directly observable.

## Introduction

Many decisions are not triggered by a single event but based on multiple sources of information. When purchasing a new computer, for instance, we certainly look at the price, but not without accounting for further aspects like quality and appearance. Usually, these multi-attribute decisions evolve sequentially, that is, as long as the collected evidence is insufficient to motivate a particular choice we search for more information to resolve our uncertainty. Importantly, such “decisions not to decide” are not directly observable but can promote significant changes in behavior (like consulting a salesman or leaving the shop).

The temporal emergence of decisions is well captured by sequential sampling models (SSMs), a mathematical approach that allows making inferences on both, how and when people decide [1]–[3]. The core structure of an SSM consists of an evidence accumulation process that proceeds until an internal criterion (a decision threshold) is met and a specific response is elicited. SSMs have a long tradition in research on perceptual decision making [4]–[6], but they also predict accuracy and response times (RTs) of preferential choices [7]–[10]. They are used to model rapid [11], [12] as well as slow decisions, which may last up to several seconds [13], [14]. However, even though the assumption of a time-consuming accumulation process implies that decisions are delayed until a threshold has been reached, an explicit decision not to decide is typically not considered by SSMs.

**Figure 1:**
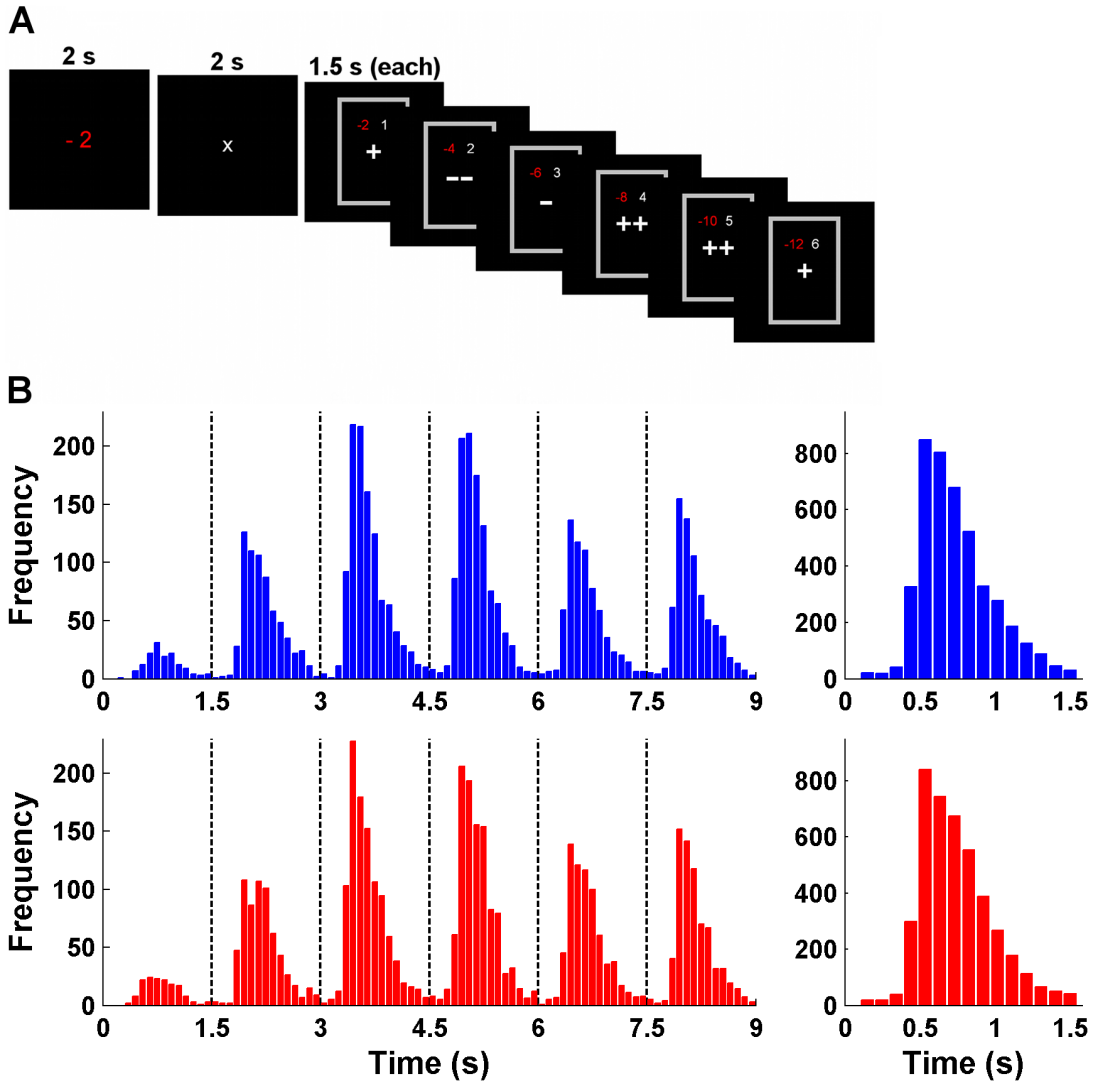
Sequential choice task and response time distributions. (**A**) Example trial of the paradigm in the EEG study. Participants were asked to either buy or reject a stock offer based on up to six sequentially presented ratings that provided probabilistic information about the stock's value. They were free to respond at any time but had to trade-off the value of collecting information against the costs for each rating. The amount of costs were presented before the first rating and varied on a trial-by-trial basis. (**B**) RT histograms for buy (blue) and reject (red) decisions summed over all participants. The left panels show data for all six ratings separately (dashed lines indicate the start of a new rating), the right panels show data summed over ratings.

Using functional magnetic resonance imaging (fMRI) and electroencephalography (EEG) in two recent studies [15], [16], we investigated the emergence of value-based decisions in the human brain. Thereto, participants performed a stock-buying paradigm (Figure 1A), in which they sampled probabilistic information (stock ratings) about stock offers in a sequential manner and were free to either buy or reject the offers at any time. We developed an SSM variant that successfully predicted which choice is made but also how many ratings are sampled. Critically, beyond the prediction of the number of sampled ratings, the model did not specify the exact timing of the decisions, that is, when exactly during the presentation of a single rating the response was made. Here, we argue that the standard SSM approach fails to account for the RT pattern in this task (Figure 1B) and presumably in many other sequential choice paradigms, as long as it does not assume the existence of a decision not to decide that can inhibit the accumulation process temporarily. We support the computational results by evidence from time-frequency analyses of the EEG data showing that the decision not to decide is accompanied by an increase in oscillatory power in the beta band (14–30 Hz), a well-established neural marker of the active inhibition of motor responses [17]–[20]. Finally, we tested our model experimentally in the context of an adapted version of our task in which the decision to sample more information is made explicit.

## Results

### Task design and response time distribution

In the following, we concentrate on the data set from the EEG study for two reasons: First, due to shorter trials and a longer test session the number of trials in the EEG paradigm was much higher than in the fMRI paradigm (320 vs. 120 trials) providing more robust parameter estimation. Secondly, in contrast to the slowly developing blood oxygen level-dependent signal of fMRI, the high temporal resolution of EEG allowed us to test for effects of the decision not to decide on oscillatory activity within single rating presentations. However, we replicate the central modeling results using the fMRI data set (see Text S1).

An example trial of the EEG task is illustrated in Figure 1A. At the beginning of the trial, participants were informed about the costs for sampling each rating during that particular trial, which were either high or low. After a short delay, the stock ratings were presented consecutively for 1.5 s each. Participants were free to respond at any time but had to decide after seeing six ratings at the latest. Positive and negative ratings determined the probability that the current stock was of positive or negative value. The goal was to buy positive and to reject negative stocks while taking the costs for sampling information into account in order to collect as many points as possible until the end of the experiment (points were then converted into real money).

The RT histograms for buy and reject decisions summed over all 27 participants are depicted in Figure 1B. The distributions for the ratings from one to six differ substantially in their absolute height and there is some variation in their shape. Nevertheless, the overall pattern is similar across ratings and comparable to RT distributions obtained from non-sequential decision-making paradigms [21]: After approximately 300 ms with almost no responses, the histograms rise steeply, peak at around 600 ms, and then decline slowly. In the following, however, we will argue that it is this putatively common RT distribution that cannot be addressed by the standard SSM approach.

### A computational model to predict response times

The SSM tested in our previous work [15], [16] was designed to predict the number of sampled ratings but did not estimate RT distributions within the time window of a rating. This is because for simplicity the model assumed that a rating presented one single piece of evidence that changes the accumulation process just once without further specifying when this change occurs. Yet, the model allowed us to explain three central findings: First, higher evidence induced earlier responses (i.e., less sampling of ratings). This was explained by conditioning the decision variable (*DV*) of the accumulation process on the log-evidence (*LE*) from already sampled ratings. Secondly, less evidence was required at later ratings. This was accounted for by a time-variant decline of the decision threshold. Thirdly, higher costs for sampling (manipulated only in [16]) also reduced the amount of sampling, which was explained by lower decision thresholds in high cost trials. Importantly, these effects were also apparent in the RT distributions, that is, higher evidence, choices at later ratings, and higher costs were associated with shorter RTs. We therefore concluded that the model is generally consistent with the observed RT effects but unable to predict them directly.

Evidently, a model that can predict RTs requires that the development of the *DV* is described with higher temporal resolution. This can be achieved by assuming that the *DV* does not change only once when a new rating is perceived but continuously (i.e., at multiple time steps during a single rating presentation). More specifically, we assume that the *DV* changes continuously depending on the accumulated *LE* for a choice option *c* (see [15] for how *LE* is calculated and updated based on sampled ratings):
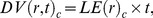
(1)where *r* is the number of the current rating (from 1 to 6 in our task) and *t* is the time elapsed during the current rating. In our case, we assumed that each time step *t* is 10 ms long (which is an arbitrary choice providing a sufficient temporal resolution), so that 150 time steps result for the duration of 1.5 s per rating. In the framework of SSM, Equation 1 basically states that the accumulated *LE* from all sampled ratings defines the current drift rate. Importantly, instead of defining one *DV* we used two separate accumulators for the two choice options (i.e., buy and reject). This allows an easy extension of the model for including a third option (i.e., the decision not to decide) in a third accumulator as described below. Furthermore we assumed that the accumulation process is noisy by adding to the *LE* an error term drawn from a normal distribution with a mean of zero and a standard deviation σ as a free parameter. Due to this noise the model makes probabilistic predictions about what and when choices are made. Our definition of a linear *DV* that varies stochastically from trial to trial (and from rating to rating) is comparable to other SSM approaches such as the LATER model [11], the Random Ray model [22], or the LBA model [23]. To derive the cumulative probability *P* that a choice has been made until *t* (within any rating *r*), the expected position of the *DV* at *t* is compared to the decision threshold θ:
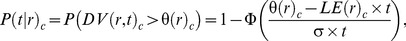
(2)where Φ(*x*) represents the cumulative normal density function at *x* (cf. [23]). The probability *p* that a choice is made at *t* is given by the partial derivative of *P* with respect to *t*:
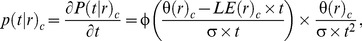
(3)where φ (*x*) represents the normal density function at *x*. However, Equation 3 only specifies the probability of a choice (e.g., buy) at *t*, given that no other choice (e.g., reject) has been made until *t*. Thus, the probability *f* of a particular choice at *t* that is not conditioned on the probability that no other choice has been made until *t* is:
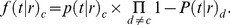
(4)So far, we have only described the model within a single rating. Across ratings, choice probabilities decline with the increasing probability of a choice at earlier ratings:
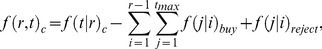
(5)where *t_max_* is the number of time points within each rating (we also included a parameter *t*_0_, representing the non-decision time within each rating, such that: *t_max_* = 150−*t*_0_). As in our previous work, we normalized the probability of choosing at the sixth rating (given that a choice has not been made before) to 1, because participants knew that they were punished for not responding at all. To account for the reduction in required evidence and mean RTs at later ratings and under high sampling costs [15], [16], we allowed the decision threshold to decrease (or increase) over consecutive ratings and to vary between high and low cost trials:
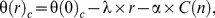
(6)where θ(0) is the initial threshold, λ controls the strength of the threshold decrease (or increase) over ratings, and α controls the influence of the costs in trial *n* on this threshold (*C* = 1 for high costs; *C* = 0 for low costs). Taken together, the model has 6 free parameters: the standard deviation σ of the error component of the *DV*, two initial thresholds for the two choice options θ(0)*_buy_* and θ(0)*_reject_*, the non-decision time *t*_0_, and the two threshold modulators α and λ. Critically, the model specifies choice probabilities for every 10 ms, allowing us to test its performance with respect to i) whether the stock was bought or rejected, ii) how many ratings were sampled, and iii) when exactly during a rating the response was made.

**Figure 2:**
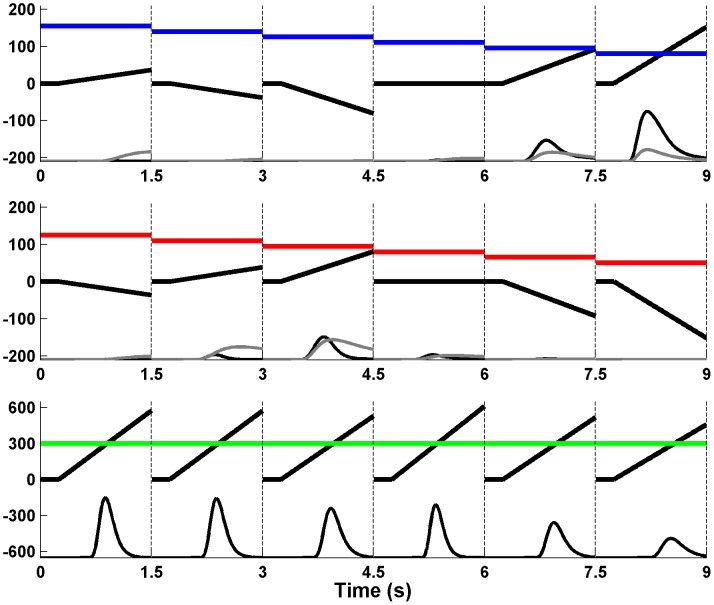
Illustration of the sequential sampling approach. The displayed model predictions refer to the example trial in Figure 1A. Each panel represents a choice accumulator over the course of the whole trial (upper panel = buy, middle panel = reject, lower panel = decision not to decide). The colored lines refer to the decision thresholds, the thick black lines to the average position of the decision variables (*DV*). The *DV* varies by Gaussian noise such that the model specifies the time point of the choice probabilistically. At the bottom of the three x-axes the model predictions for the models M1_standard_ (gray line) and M1*_evidence_ (black line) are depicted. The two models differ from each other by (not) assuming the existence of the third accumulator.

The two upper panels in Figure 2 illustrate the model described so far (for the example trial in Figure 1A). The upper panel represents the accumulation process for the buy accumulator, the middle panel for the reject accumulator. At the onset of every rating, the *DV*s are (re-)set to zero. During a rating the *DV*s then develop linearly with slopes given by the *LE* (e.g., at the first rating the *LE* is in favor of buying the stock, so *DV_buy_* moves toward the decision threshold). The thresholds decrease from rating to rating. Of note, the model accounts for effects of evidence, number of sampled ratings, and sampling costs in the same way as the model in our previous work. Furthermore, it is comparable to other established SSM approaches [11], [23].

Why should this standard model have difficulties with predicting the observed RT distribution if it does not include an explicit option of postponing decisions? A closer look at Figure 2 should clarify this point: At the first rating, for instance, *DV_buy_* moves toward the threshold such that the probability for buying the stock rises (as indicated by the gray line above the x-axis). This increase appears to be monotonic (during the 1.5 s of the first rating), which is inconsistent with the descent of the RT distribution after 800–1000 ms (Figure 1B). A similar prediction emerges for rejecting at the second and third rating. Here, the choice probability does not increase monotonically, but the descent at later time points is too shallow compared to the data. Only at the sixth rating the probability distribution (for buying) resembles the typical RT distribution. The reason for these patterns is that the probability that a choice has already been made is distributed over the entire course of the trial (i.e., over multiple ratings). At early ratings, this probability is very low and has a small impact on the actual choice probability *f*(*r*,*t*)*_c_* (see Equation 5). This is different to non-sequential tasks, in which the probability of choosing between two stimulus presentations is close to 1 (because a trial consists of only one presentation). Thus, the standard SSM does not predict a decrease in choice probability as long as the probability of an earlier choice remains low (except for the resetting of the *DV*s to 0 when a new rating occurs). In fact, the probability of earlier choices is the only explanation for the descent of RT distributions in the standard SSM approach [21]. In conclusion, the approach has difficulties with our observation that responses at late time points (1–1.5 s) within each rating presentation are extremely rare regardless of how many ratings have been sampled.

In contrast to this, we propose that participants explicitly considered not deciding at the current rating in order to wait for more information. In the context of our model, the decision not to decide is represented by a third accumulator that joins the “race to the thresholds” (lower panel in Figure 2). If this accumulator reaches its threshold before the other two, the race is stopped without an observable response, and resumed when a new rating occurs. This simple assumption which requires only one additional free parameter (the threshold for the third accumulator θ*_wait_*) helps to overcome the problem of the missing descent of choice probabilities, because the decision not to decide becomes more and more likely during the course of a single rating making choices at late time points improbable.

Importantly, the drift rate of the *DV* for the third “wait” accumulator needs to be specified. We considered three different implementations: In its simplest form, the drift rate could be a constant value (e.g., 1) such that the *DV* linearly increases in the same manner for every rating:
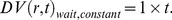
(7)Alternatively, the *DV* could depend on the number of already sampled ratings, because the utility of not deciding and waiting for more information decreases over time:
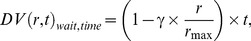
(8)where γ is a free parameter controlling the impact of the current rating number *r*, and *r_max_* is the maximum number of ratings (6 in our task) and used to standardize γ across tasks with different amount of information. Note that this implementation is equivalent to a variable threshold (therefore θ*_wait_* itself does not change over ratings, see Figure 2). Finally, the drift rate could be a function of the accumulated evidence (*LE*) for buying and rejecting so that higher total *LE* (equivalent to |*LE*(*r*)*_buy_*|, see [15]) leads to a slower rise in the *DV*, because higher evidence for deciding means lower utility of not deciding):
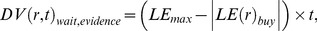
(9)where *LE_max_* is the highest log-evidence possible in the task (when the 6 ratings are either all “+ +” or “− −”), which keeps the term in parenthesis ≥0.

### Model comparison I: Predicting the number of sampled ratings

**Figure 3:**
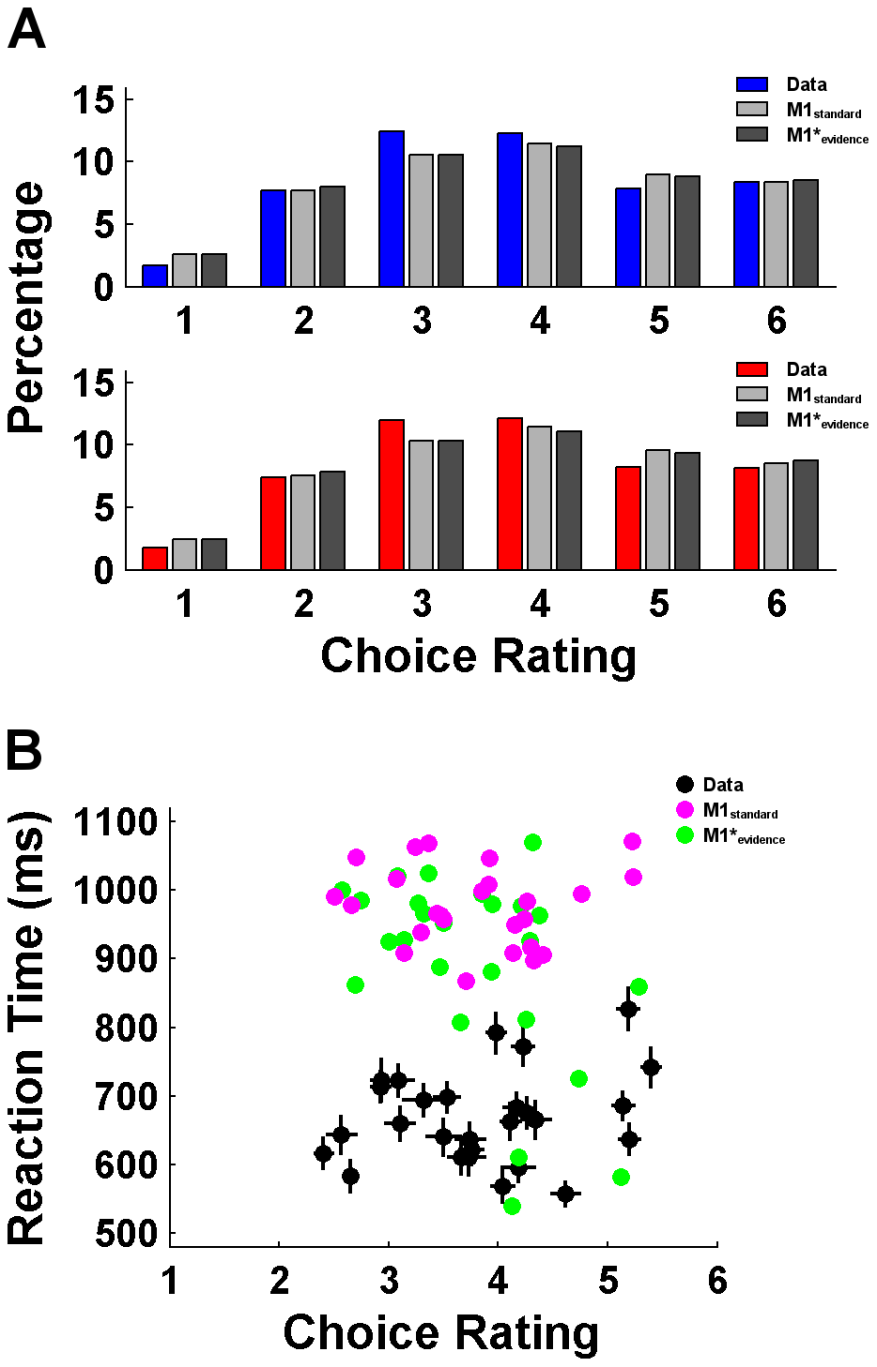
Model comparison based on the number of ratings. (**A**) Relative frequencies of buy (blue) and reject (red) decisions as well as model predictions of M1_standard_ and M1*_evidence_ when estimated to predict the number of acquired ratings, separately for the ratings from 1 to 6. (**B**) Average choice point in terms of rating number (x-axis) and mean RT (y-axis) per participant together with the respective predictions from the models M1_standard_ and M1*_evidence_. Horizontal and vertical lines at the data points represent 95% confidence intervals.

We first estimated the computational models with respect to the probability with which they predicted the observed choice (buy or reject) at the observed rating number (from 1 to 6) as realized in our previous studies [15], [16]. On the one hand, this was done to ensure that the new implementations perform roughly as well as our previous SSM. In addition, we intended to demonstrate that the new models' predictive accuracies are similar as long as the RT distributions are not considered. In terms of the Bayesian Information Criterion (BIC), the model from our previous studies (“M0”) outperforms the new models (all “M1”) (Table 1). However, the new models do a comparatively good job and predict choices (∼90%) and number of sampled ratings (∼65%) almost as accurate as M0. Most importantly, there are virtually no differences between the new candidates that include a decision not to decide (all “M1*”) and the model without this decision (“M1_standard_”) (Table 1; Figure 3A). Figure 3B shows the average choice rating (i.e., the rating at which the choice was made) and RT (i.e., the exact time of the decision within the choice rating) per participant together with the predictions from the new models M1_standard_ and M1*_evidence_. With respect to the choice ratings (x-axis) both models lie in the range of the data, but with respect to RTs (y-axis) both models predict mean RTs that are evidently too high (except for some values of the M1*_evidence_ model). This is to be expected as the models were estimated only on the basis of the choices and without using RTs. Taken together, the new models predict choices and how many pieces of information are acquired, but fitting the number of sampled ratings alone leaves open whether the assumption of a decision not to decide provides any advantage in describing the cognitive process of sequential value-based decisions.

**Table 1:** Model comparison I & II.

	Models				
	M0	M1_standard_	M1*_constant_	M1*_time_	M1*_evidence_
n (parameters)	5	6	7	8	7
Fit to rating					
BIC	673.2	711.7	713.7	713.7	704.7
	(171.7)	(168.6)	(164.9)	(158.6)	(159.8)
n (best BIC)	20	5	0	0	2
% choice	91.0	90.4	90.3	90.6	90.4
	(3.1)	(3.3)	(3.3)	(2.9)	(3.2)
% rating	66.2	64.8	65.1	65.7	64.8
	(10.2)	(10.0)	(9.9)	(9.5)	(9.8)
Fit to RT					
BIC	–	2589.3	2362.7	2320.2	2259.2
		(243.1)	(234.4)	(246.5)	(248.8)
n (best BIC)	–	0	1	6	20
% choice	–	84.7	89.1	90.3	89.8
		(5.7)	(3.3)	(3.1)	(3.0)
% rating	–	47.1	58.9	62.4	60.5
		(14.0)	(7.0)	(9.5)	(9.3)
% RT	–	11.3	18.0	18.1	18.1
		(2.6)	(5.4)	(5.6)	(6.2)

Note. n (parameters) = number of parameters per model; BIC = Bayesian Information Criterion (averaged over participants); n (best BIC) = number of participants, for which the respective model had the lowest BIC value; % choice/% rating/% RT = average percentage of trials, in which the peak of the choice probability function of the respective model matched the actual choice/number of sampled ratings/100 ms bin (chance levels are: 50.0%/16.7%/6.7%). Values in parentheses are standard deviations.

### Model comparison II: Predicting response times

**Figure 4:**
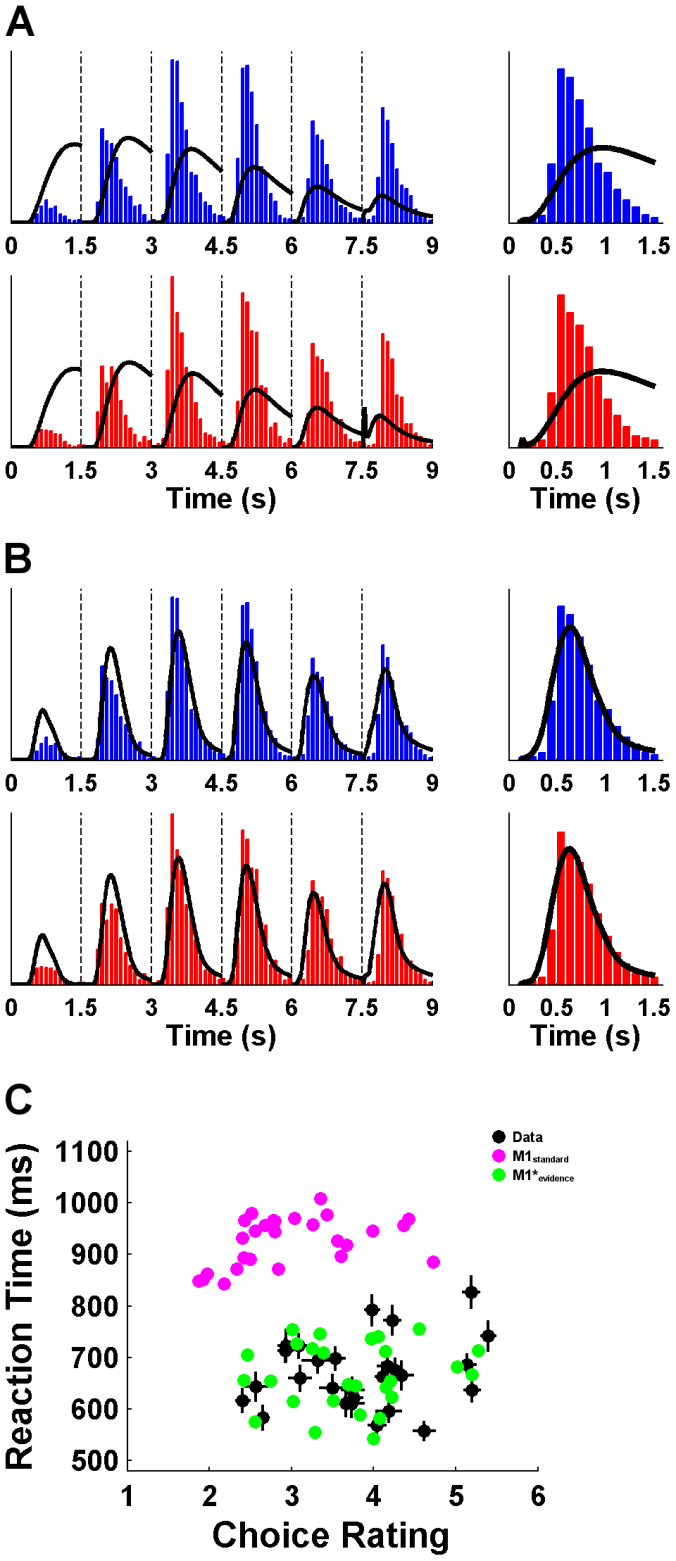
Model comparison based on response times. (**A**) RT histograms of buy (blue) and reject (red) decisions as well as model predictions of M1_standard_ when fitted to predict RTs. (**B**) Same as in (**A**) but with predictions of M1*_evidence_. (**C**) Average choice point in terms of rating number (x-axis) and mean RT (y-axis) per participant together with the respective predictions from the models M1_standard_ and M1*_evidence_.

Figure 4A and B display the RT distributions for buy and reject decisions together with the fits of models M1_standard_ and M1*_evidence_, respectively (illustrative model fits of two individuals are provided in Text S1). It is obvious that M1_standard_ performs poorly. In contrast, M1*_evidence_ reproduces the data accurately. Note that this model fits best among all candidates, but the other two models with a decision not to decide are also better than M1_standard_ (Table 1). The table also shows that only the M1* models are still able to predict the number of sampled ratings at a high level. Hence, M1_standard_ seems to fail in two different ways: First, within ratings the model underestimates the probability of early decisions and overestimates the probability of late decisions; secondly, across ratings the model overestimates the probability of choosing at early ratings and underestimates the probability of choosing at late ratings. These patterns become most evident when looking at the prediction of average choice rating and RT per participant (Figure 4C): Whereas predictions from M1*_evidence_ nicely overlap with the data, predictions from M1_standard_ are too low with respect to choice ratings and too high with respect to RTs. These specific failures are consistent with our hypothesis that a model without a decision not to decide should have difficulties with accounting for the decrease in choice probability at later time points within early ratings. In fact, M1_standard_ seems to overestimate the probability of choices at early ratings in order to “push” the probability that a choice has been made, as this is the model's exclusive feature to predict a descent in the probability function.

Interestingly, the height of the threshold of the decision not to decide (a free parameter in M1*_evidence_) correlates with the mean RT across participants (*r* = .62; *p*<.001). This is to be expected, because with a higher threshold the decision not to decide takes longer. Thus, the accumulation process is terminated later making buy and reject decisions at later time points still possible, which in turn leads to a higher mean RT value. In conclusion, comparing the models on the basis of RT predictions demonstrates the superiority of SSMs which assume the existence of an explicit decision not to decide. Moreover, the fact that M1*_evidence_ performs best suggests that the decision not to decide does not occur at a fixed time point, but that participants considered buying or rejecting the stock offer longer if evidence for one of the two choice options was high.

### Model comparison III: Alternative solutions

Beside our SSM adaptation, many other approaches allow predicting RTs in sequential choice tasks. A question warranted here is whether our realization makes the standard SSM particularly poor and whether alternative implementations would perform better – even without assuming a decision not to decide. The critical features of our solution are: i) the resetting of the *DV* to zero at every new rating, which could be replaced by a continuous *DV*; ii) the assumption that the *DV* depends on the accumulated *LE*, which could be replaced by a *DV* that depends only on the current *LE*; iii) the assumption of a linear *DV* that varies only between ratings, which could be replaced by a *DV* that stochastically varies every 10 ms; iv) the assumption of separate accumulators for each decision, which could be replaced by having a single accumulator representing the difference between the two options (i.e., a random walk with upper and lower thresholds).

**Figure 5:**
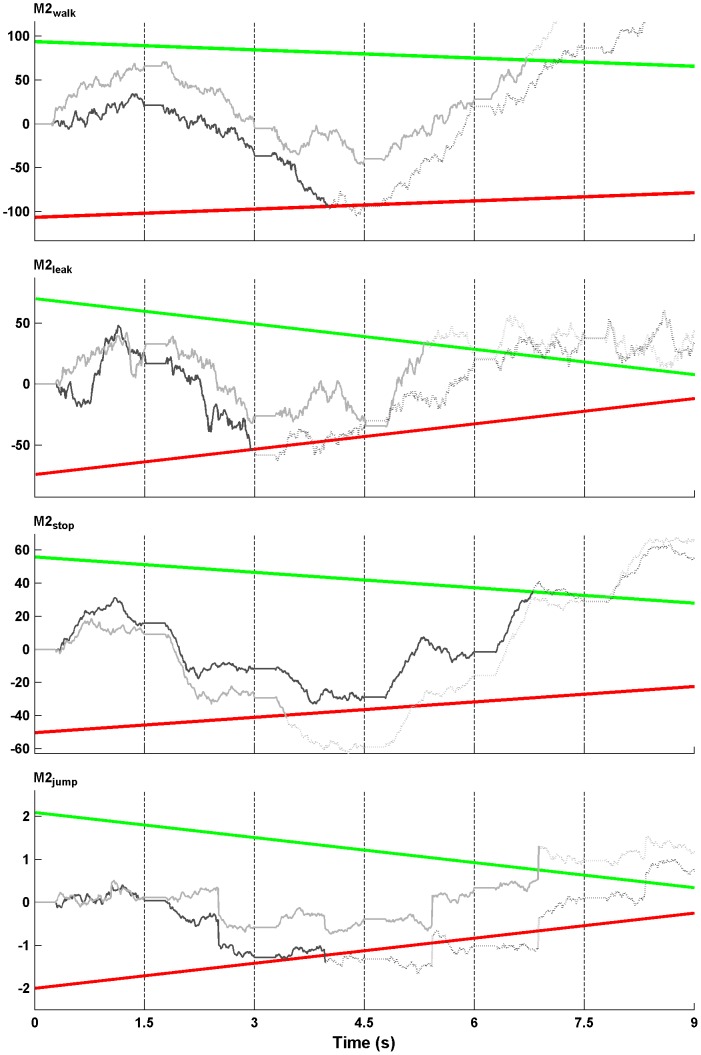
Illustration of the random walk models. For each of the four models, two separate simulations (light and dark gray) of the trial shown in Figure 1 are depicted using the parameter values of the subject that was closest to the group average (in terms of mean choice rating and mean RT). Green and red lines represent the thresholds for buying and rejecting, respectively. After crossing a threshold the simulation lines are dashed to show how the walk would have proceeded.

Hence, we tested four additional SSMs that were all based on the framework of a continuous random walk (see Figure 5 for graphical illustration and Text S1 for mathematical implementation). Note that we cannot provide a closed-from solution for these models because the position of the *DV* after the first rating (and any other rating) is not fully known. Instead, we simulated each model 100 times per trial (leading to 32,000 simulations of each model per participant) and used the frequency of correct choice and RT predictions to fit the models and to approximate log-likelihood and BIC. In addition, we also simulated M1_standard_ and M1*_evidence_ for facilitating comparisons.

Among the new candidates (all “M2”), the first model (“M2_walk_”) is the standard random walk model and most similar to M1_standard_. Here, the *DV* stochastically varies between the upper threshold for buying and the lower threshold for rejecting, and every new rating changes the drift rate of the walk. The model has the same number (and type) of free parameters as M1_standard_. The second model (“M2_leak_”) additionally assumes that the change in the *DV* depends on the current distance of the *DV* from the origin. This introduces a “leak” in the accumulation which is known as the Ornstein-Uhlenbeck process [3], [7], [8], [24]. The third model (“M2_stop_”) is similar to M2_walk_ but here the current *LE* influences the *DV* only at the beginning of each rating for a specific time period (afterwards, the *DV* fluctuates without any drift until the next rating appears). The time period, in which the current *LE* influences the *DV*, is not fixed but depends on the amount of accumulated evidence and the number of sampled ratings (see Equation S7 in Text S1). This was implemented to accommodate the influences of evidence and rating number on mean RTs as described above. In the fourth model (“M2_jump_”) the *DV* usually fluctuates without any drift but at some point (also specified according to Equation S7) the *DV* jumps up or down in dependence of the current *LE*. M2_jump_ can be regarded as a direct adaptation of the model M0 from our two previous studies [15], [16], as this model assumes that each rating changes the *DV* only once.

**Figure 6:**
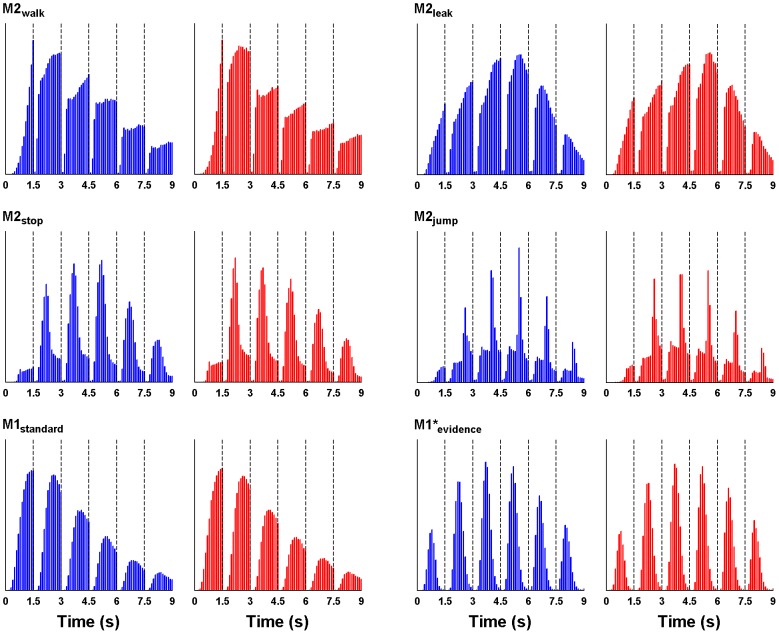
RT distributions for the simulated random walk and accumulator models. The histograms show the frequencies of simulated choice predictions (blue = buy, red = reject) per model for the ratings 1 to 6, averaged over all participants.

Figure 6 shows the simulated RT histograms of the four new models along with the simulation results of M1_standard_ and M1*_evidence_. First of all, the predictions of M2_walk_ and M1_standard_ are very similar: Both models overestimate the probability of deciding at early ratings and the probability of deciding at late time points within each rating. M2_leak_ seems to overcome the first but not the second problem. The RT histogram of M2_jump_ is very odd with very narrow and high peaks (due to the abrupt jumps in the *DV*), which is not in line with the observed RT distributions. Only M2_stop_ seems to provide an acceptable RT pattern with fast increases at the beginning and slow decreases at the end of each rating. The comparison of RT histograms is supported by a formal model comparison by means of the (approximated) BIC (Table 2): M2_walk_ and M1_standard_ perform similarly, M2_leak_ is slightly better but still much worse than M1*_evidence_. M2_jump_ has the worst fit of all models, and only M2_stop_ can compete with M1*_evidence_.

**Table 2:** Model comparison III.

	Models					
	M2_walk_	M2_leak_	M2_stop_	M2_jump_	M1_standard_	M1*_evidence_
n (parameters)	6	7	9	9	6	7
BIC	2835.4	2756.9	2350.5	3097.9	2873.9	2298.8
	(190.9)	(166.2)	(246.2)	(141.1)	(220.8)	(165.5)
n (best BIC)	0	0	10	0	0	17
% switch	55.5	54.1	54.5	52.7	88.4	86.9
	(15.7)	(15.2)	(16.4)	(10.8)	(5.2)	(6.6)

Note. n (parameters) = number of parameters per model; BIC = Bayesian Information Criterion (averaged over participants); n (best BIC) = number of participants, for which the respective model had the lowest BIC value; % switch = average percentage of model predictions matching participants' choices after a switch in evidence. Values in parentheses are standard deviations.

The comparison between the accumulator and the random walk models suggests that the poor fit of the standard approaches (M1_standard_, M2_walk_, M2_leak_) does not depend on the exact adaptation of the SSM framework to the current task. In addition to the quantitative model comparisons, however, the two classes of models can be compared by a specific different qualitative prediction: They make very different choice predictions in situations in which the accumulated evidence (*LE*) switches from favoring one choice option over the other (buy vs. reject). Here, the accumulator models (i.e., M1) predict that the majority of decisions will be made in accordance with the new evidence, because the drift rate depends only on the updated *LE*. On the other hand, the random walk models (i.e., M2) predict that decisions are approximately equally distributed across the two options, because the *DV* moves from one threshold towards the other. In fact, nearly 95% of participants' choices that were made directly after a switch in *LE* were in accordance with the updated *LE*. Consequently, only the accumulator models were able to predict these choices above chance level (Table 2). Altogether, considering the quantitative and qualitative model comparisons we conclude that M1*_evidence_ predicts choices and RTs for our sequential choice task best.

### Time-frequency analysis of EEG data

We hypothesized that the hidden decision not to decide should be detectable on the neural level. Our modeling results suggest an alternation of two processes within each rating, namely the consideration of a choice during the first half of the presentation time that is followed by its termination (or inhibition) unless a choice is really made. Therefore, we looked for a neural marker that can reflect both, preparation and inhibition of responses. Oscillations in beta-band frequencies (14–30 Hz), which are measurable via time-frequency analysis of EEG data, fulfill this criterion. More specifically, a decrease in beta power has been associated with preparation of motor responses [25]–[27] and an increase in beta power with stopping responses and maintaining the current behavioral status [17]–[20]. Hence, we predicted that for ratings without buy or reject decisions the oscillatory activity in the beta-band should decrease during the first half of the 1500 ms time window (reflecting response preparation) but then increase again (reflecting response inhibition).

**Figure 7:**
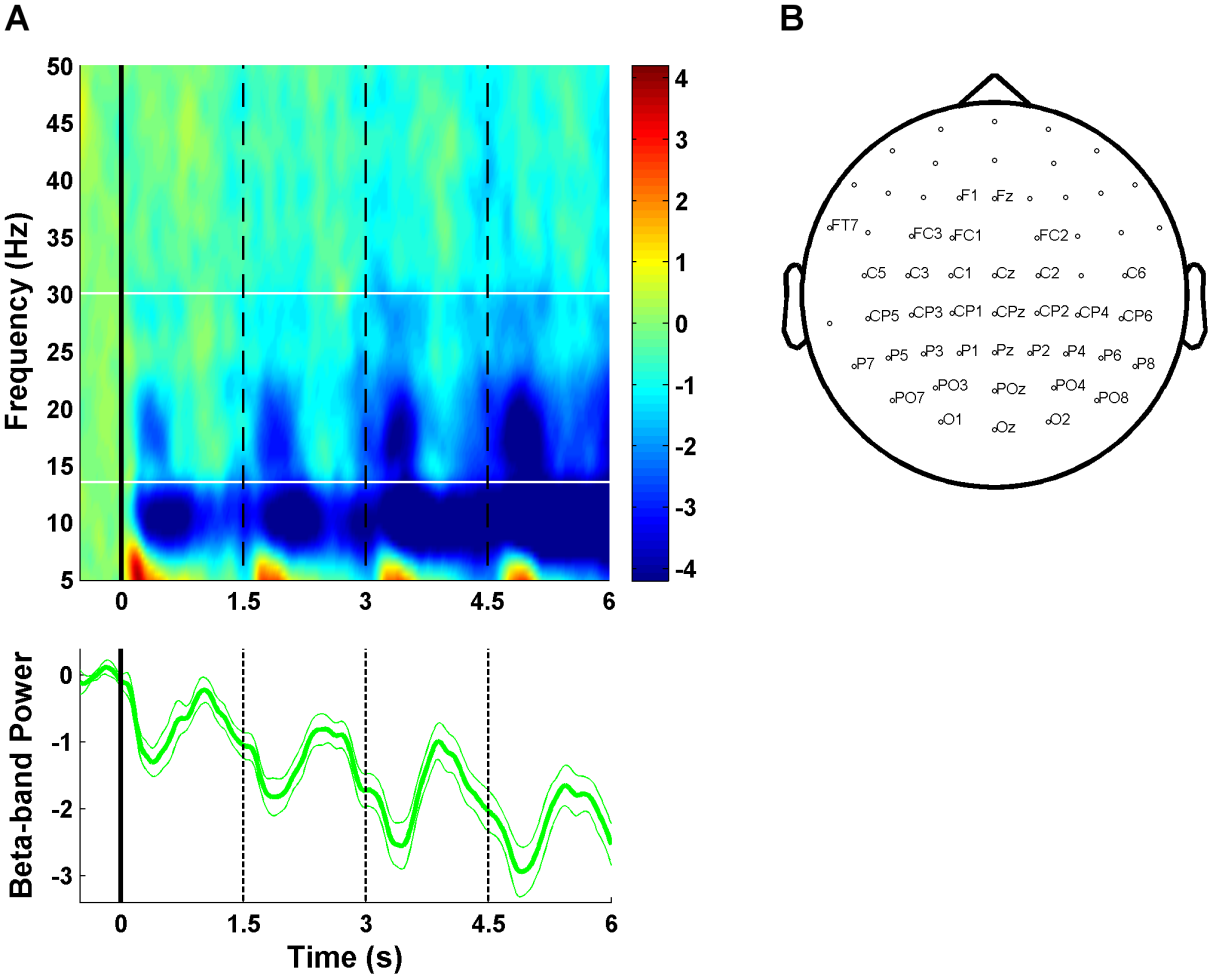
EEG time-frequency analysis. (**A**) The upper panel shows spectral power from 5 to 50 Hz averaged over all 57 scalp electrodes for the first four ratings (data for later ratings are missing for some participants, who always responded earlier), baseline corrected to the 500 ms before the first rating. The white, horizontal lines indicate the range of beta frequencies (14–30 Hz). The lower panel shows the power in the beta-band. A general negative trend in the signal and the alternation between de- and increases at every rating are clearly visible. (**B**) Position of scalp electrodes. The 37 labeled electrodes show the expected effect in the beta band (i.e., lower average signal in the first half of each rating than in the second half) at *p*<.001. Note that all EEG analyses are restricted to ratings at which no response was made.

Figure 7A (upper panel) shows the development of induced oscillatory power from 5 to 50 Hz during the first four ratings (for which we had data from all participants) averaged over all 57 scalp electrodes. First of all, the signal shows a general decrease over time that is strongest in the alpha-band (8–13 Hz) and weaker in the gamma-band (31–50 Hz). However, most important are the step-wise decreases in the beta-band at the beginning of each rating, which are followed by a recovery in power after approximately 500 ms (see also the lower panel of Figure 7A which depicts the signal average over all beta frequencies). This alternation of de- and increases at each rating corroborates our hypothesis that both the decision process and its inhibition should be reflected in beta-band oscillations. Note that the pattern was highly frequency-specific: in the beta-band it was present in 37 out of 57 channels at the statistical level of *p*<.001 (Figure 7B), but only one channel in the alpha-band (P1) and no channel in the gamma-band showed this effect at the very liberal level of *p*<.05. Notably, in the phase-locked analysis the general decrease in beta-power was also present, but the repeated de- and increases at every trial could not be observed (see Figure S4 in Text S1).

**Figure 8:**
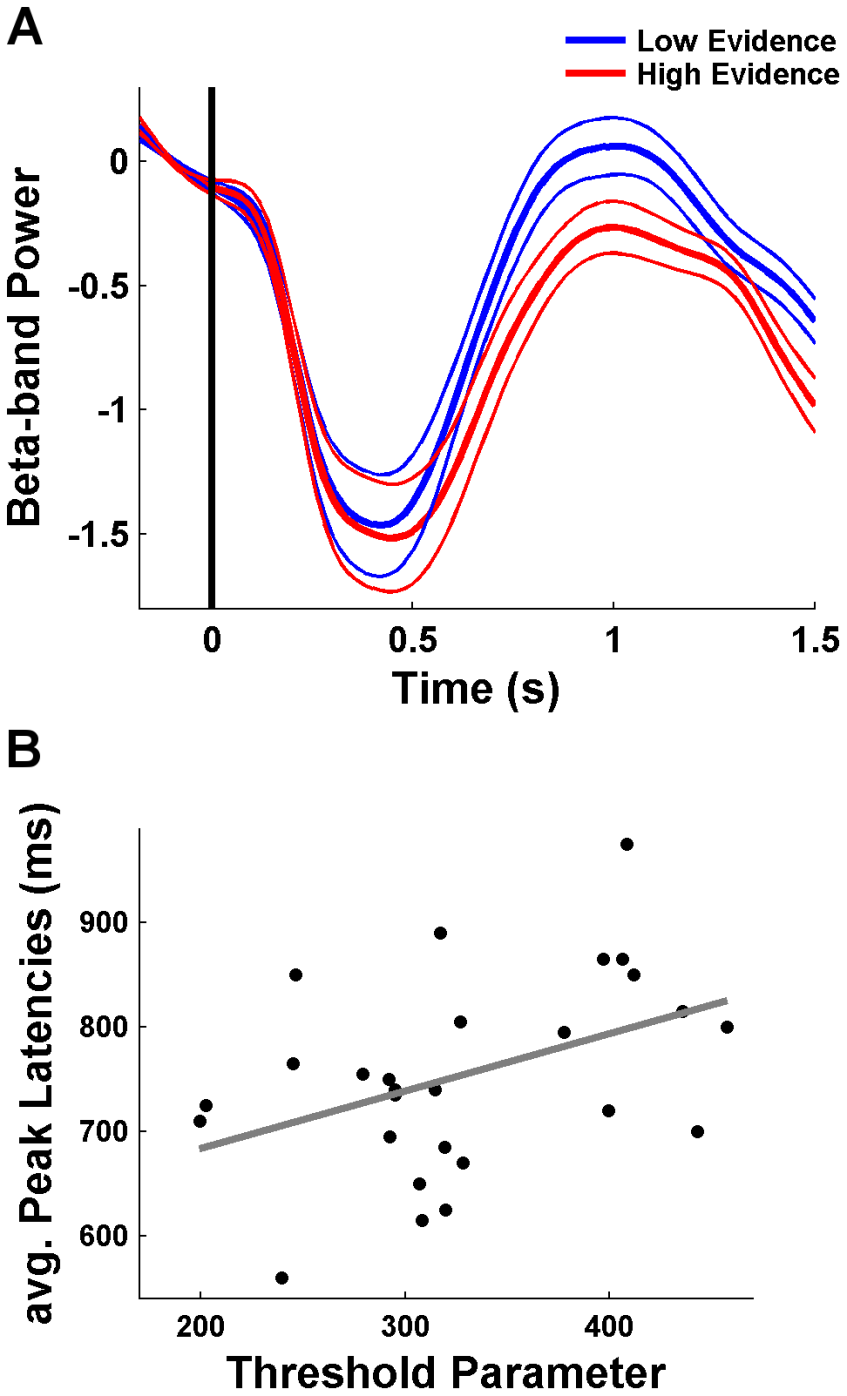
Relationship between computational and neural variability. (**A**) Beta-band power averaged over the 37 channels shown in Figure 7B separated for low and high evidence. The recovery of beta power is delayed in high evidence ratings. (**B**) The threshold parameter for the decision not to decide (according to M1*_evidence_) was correlated across participants with the averaged peak latencies for the de- and increase in beta power.

We were further interested in bringing the effects of intra- and inter-individual variability in the computational and neural data together. As stated before, the model comparison supported the M1*_evidence_ model, in which the drift rate of the decision not to decide depends negatively on accumulated evidence. This indicates that higher evidence induces a later inhibition of the choice process (within each rating). Therefore, we tested whether at ratings with high evidence the pattern of de- and increase in beta-band power is delayed. For each rating number (from 1 to 6) we conducted a median-split based on the *LE* and calculated peak latencies for de- and increase in the beta-band signal for the 37 significant channels (see Figure 7B) separately for low and high evidence ratings. As depicted in Figure 8A, the recovery of beta was indeed delayed for high evidence as compared to low evidence ratings (*t*_(27)_ = 3.15; *p* = .004). In addition, the recovery appeared to be reduced in amplitude as well. A similar prediction can be tested across participants: The higher the threshold of the decision not to decide, the longer the choice process (within the time period of one rating). Accordingly, the threshold parameter should be positively correlated with the beta-band peak latencies. This prediction was confirmed by the data (*r* = .42; *p* = .028) (Figure 8B).

### Behavioral study

**Figure 9:**
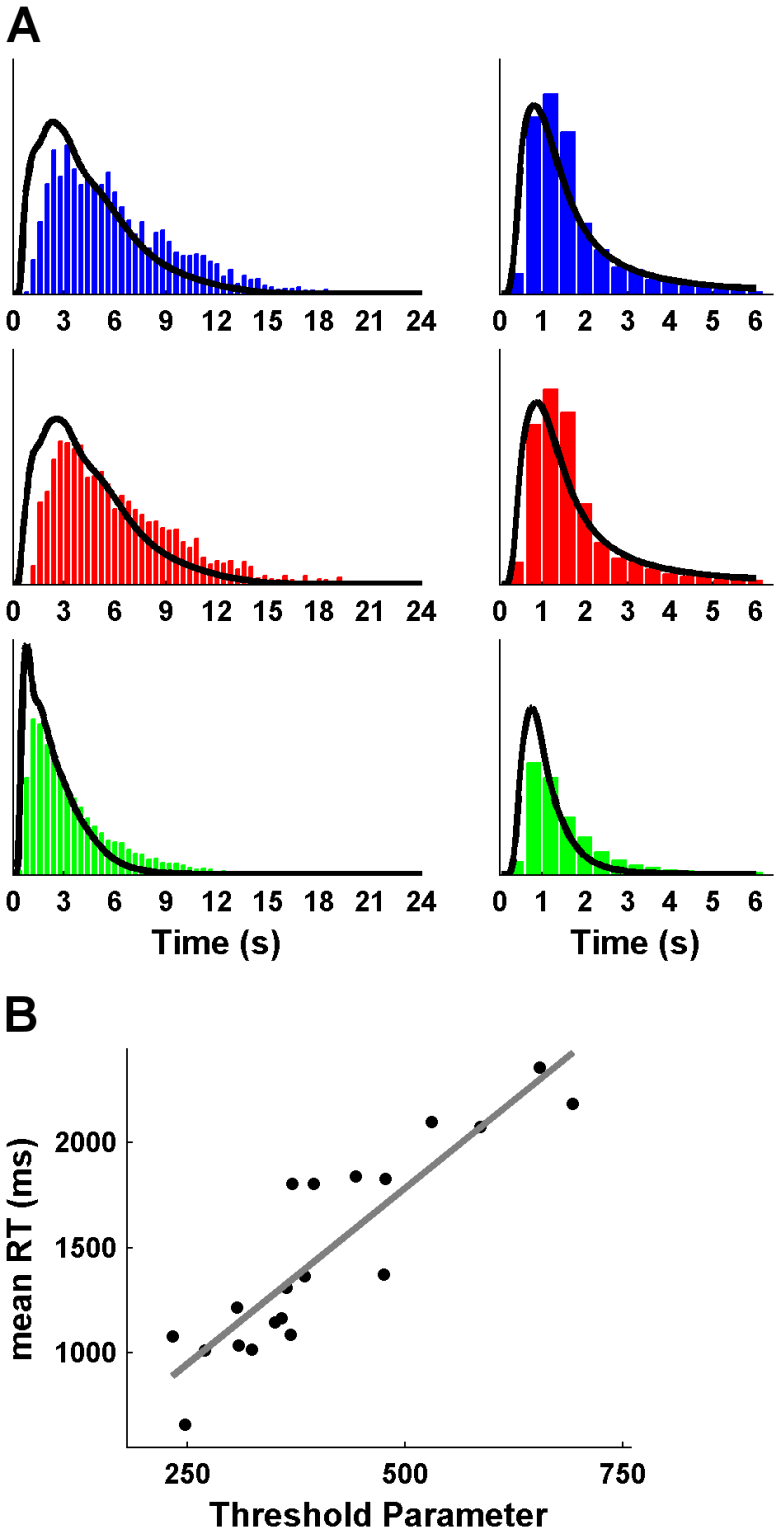
Results of the behavioral study. (**A**) RT histograms of buy (blue) and reject (red) decisions, and the decision to sample more information (green) together with model predictions of M1*_evidence_. The left panels show RTs measured from the onset of the first rating, the right panels show RTs measured from the onset of the rating at which the response was given. Note that the alternating pattern of de- and increases is washed out by the variable latency of the decision to sample more information (in contrast to the fixed duration per rating in the EEG study). (**B**) Correlation between the threshold parameter and mean RT of the decision to sample more information.

A particular strength of our cognitive modeling approach is that it revealed a hidden choice process of deciding not to decide that was not directly observable. However, the model can also be applied and generalized to choice situations in which the decision not to decide is made explicit as an observable response. Accordingly, the model should also describe RT distributions when participants have to press a button to receive a new rating and to postpone the decision. Therefore, we conducted an additional experiment with 20 participants using a behavioral task equivalent to the EEG paradigm – with the only difference being that new ratings did not appear every 1.5 s but had to be explicitly acquired via button presses (see Text S1). We used M1*_evidence_ to fit RT distributions of the decisions to buy and to reject stocks and of the decision to sample more ratings and found that the model was able to reproduce the observed RT patterns of all three types of decisions (Figure 9A) (the overall latency of buy and reject decisions is slightly underestimated due to the fact that responses at the first rating were nearly 500 ms slower than at later ratings: *t*_(20)_ = 6.67; *p*<.001). In addition, the mean RTs and threshold parameters of the decision to sample more information were positively correlated with each other (*r* = .89; *p*<.001) (Figure 9B), suggesting that the model is able to capture inter-individual differences in this adapted sequential choice task.

## Discussion

In the present study, we developed a computational model that allows making inferences on response times for a sequential choice paradigm, in which people are free to collect multiple pieces of information before they decide. The model comparison demonstrated the superiority of assuming that people actively terminate the decision process to wait for more information in this task. The time-frequency EEG analysis revealed alternating states of decreases and increases in oscillatory beta-band power supporting the view that responses are considered but then actively inhibited if the current information is insufficient to motivate a choice.

The difficulty of the standard SSM approaches in predicting the RT distribution originated from the repeated declines in choice frequency at the end of every rating presentation. In other words, the RT distribution within a rating resembled the RT pattern commonly found in non-sequential tasks but the model without a decision not to decide (M1_standard_) and the conventional random walk alternatives (M2_walk_, M2_leak_) were unable to capture the distribution over multiple ratings as a whole. An alternative solution could be to first assign the probabilities of choosing at each rating (e.g., on the basis of our model proposed in [15]) and then to model how these probabilities are distributed within ratings. However, such an approach appears psychologically implausible as it would require people to specify their choice probabilities at the beginning of each rating. But the likelihood of a choice is not simply set after 100 or 200 ms; it emerges over time, and this is what SSMs aim to model.

As stated, our SSM adaptation is just one out of many possible solutions for sequential choice tasks. However, the simulation results demonstrate that our conclusions are not restricted to the particular implementation that we chose, which is in line with the notion that different SSM approaches lead to equivalent predictions under many circumstances [3], [28]. Among the four random walk models we tested, only M2_stop_ provided an acceptable fit to the observed RT distributions. Even though this model does not include a separate accumulator for the decision not to decide, the model's concept that the influence of evidence on the *DV* is temporally restricted is very similar to the idea that the choice process is terminated at some point (and does not naturally follow from a standard random walk or diffusion model). In contrast to M1*_evidence_, however, M2_stop_ does not answer the question of why the drift rate changes during each rating. Accordingly, for tasks in which the decision to sample more information is explicitly made by the participant (as in our additional behavioral study), M2_stop_ does not provide a prediction whether and when a new rating will be sampled. In contrast, M1*_evidence_ is applicable to both scenarios and makes clear predictions that were consistent with the observed behavior. Moreover, only the accumulator models correctly predicted decisions that were made after switches in evidence. Finally, the higher mathematical feasibility of our approach, which offers a closed-form solution to modeling RT distributions in sequential choice paradigms, is a strong advantage.

Under which circumstances is it necessary to extend an SSM by including a decision not to decide? Certainly, the majority of (non-sequential) decision-making paradigms with a continual input of information can be successfully modeled without this component. However, in many decision problems faced in everyday life, people do not receive information automatically but actively and explicitly search for information. This implies that people have to decide whether the information acquired so far is sufficient to reach a decision or whether additional information should be searched for. Accordingly, there are many experiments using sequential delivery of information, particularly in the emerging field of decision neuroscience [14]–[16], [26], [27], [29]–[34] (methods such as fMRI and EEG also require well-controlled stimulus presentations). Accurate computational modeling of the decision mechanisms in sequential choice is therefore highly desirable. In addition, there is a substantial body of research on choice deferral [35], [36] where decision makers are explicitly provided with the option to refuse a choice (similar to our behavioral study). Here, the decision not to decide is relevant as it is defined as an explicit option in the decision process.

Interestingly, the assumption of a decision not to decide seems to improve the model fit in the fMRI data set even more than in the EEG data set (see Table S1 in Text S1). This could be related to the longer presentation times of ratings (2–4 s instead of 1.5 s), such that the periods are prolonged, in which participants terminate the choice process to wait for more information. Future studies should identify the conditions, under which a decision not to decide becomes particularly relevant to account for RT distributions in sequential choice. We propose that factors like the number of cues (and its variability) and the presentation time of cues (and its variability) influence for how long people postpone their decision process.

In a previous paper that was based on the same EEG data set [16], we looked at event-related potentials (ERPs) in the motor system, such as the readiness potential, to investigate the emergence of value-based decision in the human brain. The ERP analyses already pointed to the existence of a decision not to decide, insofar as the readiness potential declined after 700 ms within every rating. However, this effect provides only indirect evidence as compared to the increase in beta-band power, which has been directly linked to active response inhibition [17]–[20]. Furthermore, unlike the latency of de- and increases in the beta-band (Figure 8), we did not find any relationship between the latency of the readiness potential and intra- or inter-individual variability of the decision not to decide. A probable reason for this difference is that in contrast to ERPs, induced oscillatory signals do not have to be phase-locked to the onset of stimulus events so that they are more likely to reveal the existence and the temporal extent of endogenous brain activity [37]. In line with this, the alternating pattern in the beta-band could not be observed in the phase-locked time-frequency analysis. Finally, it should be noted that our conclusion about the existence of a decision not to decide does not rest upon reverse inference [38]: Instead of first finding the beta synchronization and then speculating about inhibition processes, we first established computational evidence for a hidden choice mechanism and then hypothesized (and corroborated) that this mechanism is reflected in a specific oscillatory EEG pattern.

Our findings demonstrate that a thorough modeling of behavioral data can reveal hidden dynamics in cognition that would remain hidden if only observable decisions are taken into account or if models are fitted against too coarsely averaged data [39]. Whereas comparing the SSM variants only on the basis of how many ratings are sampled failed to reveal the necessity of assuming a decision not to decide, the comparison based on RT distributions was decisive. Moreover, we were able to specify the decision not to decide in more detail by showing that people are faster in reaching this decision as long as evidence for either choice option is low. Our study further advocates combining computational modeling with brain imaging techniques, as both methodologies attempt to look into the “black box” of the human mind. In this context, the temporal precision of EEG nicely dovetailed with the fine-grained RT modeling allowing us to discover a decision that would otherwise not have been observable.

## Materials and Methods

### Participants

For the EEG study [16], we had data sets from 28 participants (details on materials and methods of the fMRI study [15] and the behavioral study are provided in the Supporting Information). Participants were right-handed healthy persons with normal or corrected-to-normal vision. Data of one participant, whose average RT was 3.4 standard deviations higher than the group average, was excluded. Therefore, the final sample comprised 27 participants (mean age = 25.8 years, ±3.2, 20–32 years; 14 females). The study was approved by a local ethics committee (“Ethik-Kommission der Ärztekammer Hamburg”) and accords to the principles expressed in the Declaration of Helsinki. All participants gave written informed consent. Participants were reimbursed for participation and could earn additional money by winning points in the task (every collected point was rewarded with 0.01 Euro).

### Experimental design

In the sequential decision problem, participants had to decide whether to buy or reject stock offers based on ratings (of fictitious stock rating companies) that provided probabilistic information about the stocks' value (Figure 1A). Each trial started with the cost phase (2 s), in which the participant was informed about the costs for observing one rating in that particular trial. Rating costs were either low (−2 points) or high (−5 points) according to a randomized order, presented in red in the middle of the black display screen. A break of 2 s followed, in which a white “x” was shown. Afterwards, the decision phase commenced with presentation of the first rating (1500 ms for each rating). Enclosed by a gray frame, the rating appeared (in white) in the middle of the screen and the cumulative costs for observed ratings were presented (in red) above it together with the number of observed ratings (in white). All information was presented in a narrow range in the middle of the screen to prevent excessive eye movements (screen size: 19 inches; distance to screen: ∼95 cm; horizontal visual angle: ∼1.6°; vertical visual angle: ∼2.4°). After the participant's response or the termination of the sixth rating, the next trial started with a variable delay of 2 to 6 s. Stimulus presentation was realized using the Presentation Software package (Neurobehavioral Systems).

Participants were told that stocks were either *good* (value: +80 points) or *bad* (value: −80 points) and buying a stock would lead to the payment of its value. Participants were instructed to respond whenever they wanted during the decision phase but not later than at the sixth rating (they would otherwise receive the negative value of a *bad* stock while paying the costs for all six ratings). They were further informed about the possible rating values (i.e., “− −”, “−”, “+”, or “+ +”), the cost conditions, the independence of subsequent ratings from each other, that all ratings were equally important, that the prior probability of *good* and *bad* stocks was equal, and that the ratings contained probabilistic information about stock values: They were instructed that the probability of being offered a *good* stock is increased to approx. 57% given a “+” and decreased to approx. 43% given a “−”, that “+ +” (“− −”) ratings are equivalent to two separate “+” (“−”) ratings, and that in general the more “+”s and the less “−”s are presented, the higher the probability of a good stock. In fact, the probability of a good stock is updated with every new rating according to Bayes' theorem (details are provided in [15]). Responses were made with left and right index fingers for pressing keys “X” and “M” on the keyboard, respectively. The assignment of choice options (buy, reject) and index fingers was counterbalanced across participants. Overall, 320 stocks were offered during the EEG experiment (with a break of approximately 5 minutes after 160 stocks). Rating configurations for these stocks were generated randomly for each participant with the restriction that each of the four possible rating values of the first rating was presented in exactly 25% of the trials. The length of the experiment depended on the amount of awaited ratings but did not exceed 90 minutes. Participants knew that the number of stocks was fixed and that faster play would not lead to getting more offers. Before the EEG experiment, the task was practiced in two runs of 40 trials; the first run included feedback about the correctness of the choice to familiarize participants with the probabilistic nature of rating information; in the second run feedback was omitted as in the main experiment.

### Model estimation

Parameter values for the computational models (see Results) were estimated using the simplex search algorithm [40] as implemented in Matlab. The algorithm was set up to find the parameter set Θ that minimized the *deviance* of a model, which is based on the log-likelihood of the behavioral data given the model [39]. If *c* is the choice (buy or reject) made at rating *r* (1–6) and time *t* (1–150 in steps of 10 ms), then the *deviance* of the model when fitted to the number of ratings becomes:
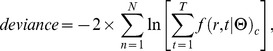
(10)where *N* is the number of trials and *T* is the number of time steps. Thus the model predictions *f*(*r*,*t*)*_c_* (see Equation 5) are summed over the 150 time steps within a rating. To fit RT distributions, this summation could simply be omitted. However, we still summed over 10 consecutive time steps (thus getting 15 bins of 100 ms length per rating), which was necessary to obtain robust modeling results. Note that our method of fitting RTs retains the information about choices and number of sampled ratings thus ensuring that a model that can predict RT distributions but not choices or number of sampled ratings would perform poorly. For model comparison the Bayesian Information Criterion (BIC) was calculated, which takes the model complexity by the number of free parameters *k* into account to penalize model complexity [39]:
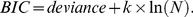
(11)


### EEG data acquisition

EEG data was recorded from 57 active Ag/AgCl electrodes mounted on an elastic cap (actiCAP, Brain Products) at standard scalp locations (Fp1, Fpz, Fp2, AF7, AF3, AFz, AF4, AF8, F7, F5, F3, F1, Fz, F2, F4, F6, F8, FT7, FC5, FC3, FC1, FC2, FC4, FC6, FT8, C5, C3, C1, Cz, C2, C4, C6, TP7, CP5, CP3, CP1, CPz, CP2, CP4, CP6, P7, P5, P3, P1, Pz, P2, P4, P6, P8, PO7, PO3, POz, PO4, PO8, O1, Oz, O2) at a sampling rate of 500 Hz. 7 additional electrodes were used to record vertical and horizontal eye movements (electrooculogram, EOG) and muscle activity (electromyogram, EMG) of two face muscles (corrugator supercilii, zygomaticus). For vertical EOG, the difference between the signal at Fp1 and an electrode positioned below the left eye was used. For horizontal EOG and EMG, the difference between the two relevant additional electrodes was used (horizontal EOG: left and right temples; EMG: two electrodes positioned over the respective muscle). Two further electrodes were used as reference (positioned at FCz) and ground (positioned at the neck). Electrode resistances were kept below 10 kOhm.

### EEG data preprocessing and analysis

EEG data preprocessing and analysis was conducted using Fieldtrip [41]. EEG data from all electrodes were first segmented into single trials with epochs lasting from 1 s before the cost phase to 1 s after the decision phase and downsampled to 200 Hz. The signal of the 57 scalp electrodes were then re-referenced to average, while EOG and EMG signals were calculated as described above. Epoch data was baseline corrected to the 1 s time window preceding the cost phase and then filtered using a high pass filter of 1 Hz and a low pass filter of 80 Hz. The long trial duration led to numerous eye blinks within trials and we thus chose to subtract blink-related and other eye movement artifacts using the Gratton-Coles algorithm [42] rather than simply deleting any trials with blinks. Artifact correction further included automatic detection and elimination of trials with excessive absolute EEG signals or excessive changes in the EEG signal. Finally, the data was inspected manually for any remaining artifacts and trials were deleted accordingly. Taking trials without responses and trials with artifacts together, data from 7.1% of the trials on average could not be analyzed.

For the time-frequency analysis, separate epochs for each rating were generated excluding those ratings at which a response was given (to avoid confusion with motor-evoked signals). Spectral power for frequencies from 5 to 50 Hz in steps of 0.5 Hz were then calculated for each rating in every trial using the “Multitapering” method for transforming time-frequencies [43] with a single Hanning taper over time windows of 400 ms in steps of 40 ms. For the phase-locked analysis, the waveforms of individual ratings epochs were first averaged and spectral power analysis was then conducted on these average waveforms [37]. To test for effects of accumulated evidence, we split epochs for every rating (from 1 to 6) into equal numbers of low and high evidence ratings (defining low and high evidence ratings in this way avoids confusing evidence- and time-dependent effects, because evidence tends to be higher at later ratings). Raw spectral data was log-transformed to obtain approximately normally distributed values for parametric analyses [44]. To show the development of time-frequency results over multiple ratings (Figure 7A), data from consecutive ratings were concatenated (but only for ratings 1–4, as some participants never sampled all information). For statistically testing the hypothesis that beta-band power first de- and then increases during a rating, we calculated the average power in the beta-band for the first and second 750 ms of each rating separately and then tested whether the power in the second half was higher than in the first half (at *p*<.001). Only channels that survived this criterion were included in the peak latency analysis (to ensure that peak latencies are estimable at all). Peak latencies were derived by measuring the time point between 250 and 750 ms at which beta power was lowest and the time point between 750 and 1250 ms at which beta power was highest (peaks before 250 and after 1250 ms were considered unrealistic). The two latencies were then averaged to obtain a single index per participant.

## Supporting Information

Text S1 Supporting information including descriptions of the random walk models and the simulations, materials and procedures of the fMRI and the behavioral study, Table S1, and Figure S1–S4.
(DOC)
