# Correction: Deciding Not to Decide: Computational and Neural Evidence for Hidden Behavior in Sequential Choice

**DOI:** 10.1371/journal.pcbi.1005476

**Published:** 2017-04-11

**Authors:** Sebastian Gluth, Jörg Rieskamp, Christian Büchel

In the Results section, there is an error in Equation 5. Please view the complete, correct equation here:

Corrected version of Equation 5:

$$f{\left(r,t\right)}_{c}=f{\left(t|r\right)}_{c}\times \left(1-\sum_{i=1}^{r-1}\sum_{j=1}^{t_{max}}f{\left(i,j\right)}_{buy}+f{\left(i,j\right)}_{reject}\right)$$

Original, uncorrected version of Equation 5:

$$f{\left(r,t\right)}_{c}=f{\left(t|r\right)}_{c}-\sum_{i=1}^{r-1}\sum_{j=1}^{t_{max}}f{\left(j|i\right)}_{buy}+f{\left(j|i\right)}_{reject}$$
