# Supplementary material for: Deciding Not to Decide: Computational and Neural Evidence for Hidden Behavior in Sequential Choice
Source: PLoS Comput Biol. 2013 Oct 31;9(10):e1003309. doi: 10.1371/journal.pcbi.1003309 (PMC3814623; doi:10.1371/journal.pcbi.1003309)
Supplement: Text S1 — Supporting information including descriptions of the random walk models and the simulations, materials and procedures of the fMRI and the behavioral study, Table S1, and Figure S1–S4. (DOC) [file pcbi.1003309.s001.doc]

**Deciding not to Decide: Computational and Neural Evidence for Hidden Behavior in Sequential Choice**

Sebastian Gluth, Jörg Rieskamp, Christian Büchel

**Supporting Information – Text S1**

Simulations of the random walk and accumulator models

In contrast to the accumulator models such as M1_standard_ or M1*_evidence_, the *DVs* of the continuous random walk models are not reset to zero at every new rating. Therefore, the expected position *E* of the *DV* at the second rating can only be probabilistically specified:

$E\left[DV\left(r=2\right)\right]=\int_{-\infty}^{\infty} \varphi\left(\frac{x-\text{δ}\times t_{\max}}{\text{σ}\times t_{\max}}\right)dx$, (S1)

where δ and σ are the drift rate and the standard deviation of the diffusion process, respectively. To get the expected position of the *DV* at the third rating, one has to calculate the distribution of expected positions at the third rating given each possible position at the second rating and to weight these distributions by the probability of each position at the second rating:

$E\left[DV\left(r=3\right)\right]=\int_{-\infty}^{\infty} \varphi\left(\frac{y-x-\text{δ}\times t_{\max}}{\text{σ}\times t_{\max}}\right)dy\times\int_{\text{θ}_{reject}}^{\text{θ}_{buy}} \varphi\left(\frac{x-\text{δ}\times t_{\max}}{\text{σ}\times t_{\max}}\right)dx$. (S2)

Note that the integral for the second rating is restricted to the space between the lower and upper decision thresholds: If the *DV* was beyond these points, a decision would have been made already. To get the expected position of the *DV* at the fourth, fifth, and sixth rating, the calculations become increasingly complex. Moreover, the decrease of the decision thresholds and the non-linearities in the *DV* definition of the models M2_leak_, M2_stop_, and M2_jump_ make the necessary computations even more complicated and a close-form solution impossible (note that for obtaining RT predictions one would need to estimate *E*[*DV*] not only for the start of each rating but also for every time point within each rating).

Thus, we decided to approximate the models’ predictions by simulating them (see Figure 5). Common to all simulated models is that the *DV* is fixed for an initial time period reflecting the non-decision time *t*_0_. This can be expressed defining the change in the *DV* as 0:

$\Delta DV=0$. (S3)

After *t*_0_, the *DV* of the basic random walk model M2_walk_ drifts according to the log-evidence of the current rating *cLE*(*r*) and is also affected by noise *e* which is sampled (every 10 ms) from a normal distribution with mean = 0 and standard deviation = σ:

$\Delta DV=cLE\left(r\right)+e$. (S4)

In contrast, the change in the *DV* of the Ornstein-Uhlenbeck model M2_leak_ can be influenced by its current distance to the neutral point 0 [1–4]:

$\Delta DV=\text{κ}\times DV+cLE\left(r\right)+e$, (S5)

where κ is a free parameter that controls the influence of the *DV*’s position on its change (for κ < 0, the change is slower the more the *DV* is away from 0). The *DV* of M2_stop_ changes according to Equation S4 but only up to a certain point *t*_1_ at which the influence of *cLE* disappears and the *DV* fluctuates randomly until the next rating:

$\Delta DV=e$. (S6)

The time point *t*_1_ is not defined arbitrarily but accords to a function of the (absolute) accumulated evidence and the number of already sampled ratings:

$t_{1}=\text{β}_{0}+\text{β}_{1}\times\left|LE\left(r\right)\right|+\text{β}_{2}\times r$, (S7)

where β_0_, β_1_, and β_2_ are free parameters modeling the intercept of *t*_1_, and the influences of evidence and rating number on *t*_1_. The goal of linking *t*_1_ to the accumulated evidence and the number of sampled ratings was to accommodate their influences on the mean RTs (see main text). The time point *t*_1_ is also defined for M2_jump_ (albeit with potentially different parameter values), but this model assumes that the *DV* fluctuates between *t*_0_ and *t*_1_ and *t*_1_ and *t*_max_ according to Equation S6 and changes only once (at *t*_1_) according to Equation S4 (hence, the decision thresholds of M2_jump_ are much lower than in the other models; see Figure 5). Given that M2_jump_ assumes that each rating affects the *DV* only once, it can be regarded as a direct adaptation of the model M0 from our two previous studies [5,6].

Each model predicts a particular decision (buy or reject) as soon as the respective decision threshold θ* *has been crossed. We adapted the decrease in decision thresholds over time from an abrupt change at every new rating (see Equation 6 in the main text) to a continuous linear decline throughout every rating to avoid that the models predict (too) many threshold crossings at the start of new ratings:

$\text{θ}\left(r,t\right)_{buy}=\text{θ}\left(0\right)_{buy}-\text{λ}\times\left(t+t_{\max}\times\left(r-1\right)\right)-\text{α}\times C\left(n\right)$, (S8)

$\text{θ}\left(r,t\right)_{reject}=\text{θ}\left(0\right)_{reject}+\text{λ}\times\left(t+t_{\max}\times\left(r-1\right)\right)+\text{α}\times C\left(n\right)$. (S9)

Note that the influences of time and rating costs on θ_reject_ are positive because θ_reject_ has a negative sign in random walk models.

To fit the models to the data and to calculate the BIC, we approximated the log-likelihood by simulating each trial 100 times and using the frequency of correct model predictions (i.e., how often the simulation made the actual choice at the actual rating number within 100 ms of the actual RT) as a proxy for the likelihood of the data. For example, if a buy decision was made at the 3^rd^ rating at 725 ms, and 25 of the 100 model simulations made a buy decision at the third rating between 675 and 775 ms, then the log-likelihood of the model for this trial was defined as ln(.25) = –1.386. If no simulation made such a correct prediction, then the log-likelihood for the trial was simply set to ln(.001) = –6.908. Note that for simulating M1_standard_ and M1*_evidence_ we used the same definition of noise *e* and decision thresholds θ as well as the same approximation of the log-likelihood to maximize commensurability with the random walk models.

Participants, design, and model fitting of the fMRI study

Participants of the fMRI study [5] were right-handed healthy persons with normal or corrected-to-normal vision. The sample comprised 26 participants (mean age = 25.4 years, ±3.6, 21–36 years; 12 females). The study was approved by a local ethics committee ("Ethik-Kommission der Ärztekammer Hamburg") and accords to the principles expressed in the Declaration of Helsinki. All participants gave written informed consent. Participants were reimbursed for participation and could earn additional money by winning points in the task (every collected point was rewarded with 0.01 Euro).

The sequential choice task in the fMRI study was very similar to the EEG paradigm (the two main differences are the absence of different cost conditions and the longer duration of rating presentations – see below). In each trial, participants were offered a stock and had to decide whether to buy or reject the offer (see Figure S1A). A gray frame enclosing the heading “offering” and the names of six fictitious rating companies were presented on the screen throughout the entire fMRI experiment. In addition, a counter at the upper left side of the frame depicted the costs for awaiting rating information and was set to a white-colored “0” between trials. A trial commenced with the rating of the first company appearing next to the first company’s name (in the EEG task, the word “offering” and the company names were omitted to reduce eye movements). Positive ratings were colored green, negative ratings were colored red. With the start of the first rating the counter turned to a red-colored “–2” (each rating cost 2 points). After a variable delay (2, 2.5, 3, 3.5, or 4 s) the second rating was displayed next to the second company’s name, the counter turned to “–4”, and the previous rating disappeared. This procedure continued until the last rating was presented (again for at least 2 s) or a response was made, which terminated the trial (i.e., the current rating disappeared and the counter was set back to “0”). Trials were separated by a variable interval of 2 to 9 s. The order of rating companies was fixed from top to bottom. Overall, 120 stocks were offered during the fMRI experiment. The length of the experiment depended on the amount of awaited ratings but did not exceed 40 minutes. Two practice sessions of 32 trials each preceded the fMRI experiment; the first session included feedback about the correctness of the choice, the second session (and the main task in the scanner) did not include this feedback. Participants received the same information about the rules of the task (e.g., stock values, probabilities) as in the EEG study.

We fitted the same set of models to the behavioral data of the fMRI study as for the EEG study. However, the fitting procedure was slightly different due to the different timing of ratings: In the fMRI task, rating presentations varied between 2 and 4 s (see above) instead of the constant 1.5 s in the EEG task. Therefore we used time steps of *t* = 25 ms (instead of 10 ms) to define the *DV* (see Equation 1) and fitted RT distributions using 16 bins of 250 ms per rating (instead of 15 bins of 100 ms). Note that we had only 120 trials for each participant, a very low number for fitting RTs, making this rather coarse procedure necessary to achieve sufficient robustness. To manage the variable rating durations, we decided to assume a choice process over 4 s at every rating. This was required because if we had assumed only 2 s per rating, the models’ probabilities for choices after 2 s (approx. 4% of all choices) would have been impossible to estimate.

Participants, design, and model fitting of the behavioral study

Participants of the behavioral study were healthy persons with normal or corrected-to-normal vision. The sample comprised 20 participants (mean age = 26.2 years, ±7.5, 20–52 years; 11 females). All participants gave written informed consent. Participants were reimbursed for participation and could earn additional money by winning points in the task (every collected point was rewarded with 0.015 Swiss Franc).

The design of the task was exactly the same as in the EEG study (e.g., same number of trials and training trials, same inter-trial intervals, same reward schedule), while the only difference being that a rating was not replaced by the next rating after 1.5 s but appeared on the screen as long as the participant did not press a button. The participants were asked to either buy or reject the stock using the buttons “X” or “M” on the keyboard or to sample a new rating using the “SPACE” button on the keyboard.

To fit M1*_evidence_, we followed the procedure of the EEG study as described in the main text with two modifications: First, because decisions were not restricted to 1.5 s anymore, we extended the time window for a single rating to 10 s and excluded all trials from the analysis in which at least one rating was presented for more than 10 s (2.2% on average). Secondly, we extended the calculation of the deviance (Equation 10 in the main text) by including the decision to sample more information (i.e., the choice *c* could either be “buy” or “reject” or “sample a new rating”):

$deviance=-2\times\sum_{n=1}^{N} \ln\left[\sum_{t=1}^{T} \sum_{r=1}^{R} f\left(r,t|\Theta\right)_{c}\right]$. (S10)

Thus, for every trial we did not obtain only one deviance value but *R* = 1 to *R* = 6 values, depending on how often the participant decided to sample more information.

**References**

1. Uhlenbeck GE, Ornstein LS (1930) On the theory of Brownian motion. Physical Review 36: 823–841.

2. Busemeyer JR, Townsend JT (1993) Decision field theory: a dynamic-cognitive approach to decision making in an uncertain environment. Psychol Rev 100: 432–459.

3. Usher M, McClelland JL (2001) The time course of perceptual choice: the leaky, competing accumulator model. Psychol Rev 108: 550–592.

4. Bogacz R, Brown E, Moehlis J, Holmes P, Cohen JD (2006) The physics of optimal decision making: a formal analysis of models of performance in two-alternative forced-choice tasks. Psychol Rev 113: 700–765.

5. Gluth S, Rieskamp J, Büchel C (2012) Deciding when to decide: time-variant sequential sampling models explain the emergence of value-based decisions in the human brain. J Neurosci 32: 10686–10698.

6. Gluth S, Rieskamp J, Büchel C (2013) Classic EEG motor potentials track the emergence of value-based decisions. Neuroimage 79: 394–403.

**Table S1. Model comparison for the fMRI data set.**

|  | Models | | | | |
| --- | --- | --- | --- | --- | --- |
|  | M0 | M1_standard_ | M1*_constant_ | M1*_time_ | M1*_evidence_ |
| n (parameters) | 4 | 5 | 6 | 7 | 6 |
| Fit to rating | | | | | |
| BIC | 272.0 (67.1) | 277.4 (69.3) | 285.2 (71.8) | 283.6 (71.5) | 279.1 (68.4) |
| n (best BIC) | 16 | 5 | 0 | 2 | 3 |
| % choice | 90.0 (4.1) | 90.0 (4.3) | 90.0 (4.1) | 90.1 (4.4) | 89.6 (4.3) |
| % rating | 62.6 (9.7) | 65.0 (9.9) | 64.8 (9.6) | 65.2 (9.8) | 63.8 (8.5) |
| Fit to RT | | | | | |
| BIC | – | 1228.8 (132.5) | 854.3 (122.2) | 878.1 (115.3) | 838.0 (139.9) |
| n (best BIC) | – | 0 | 8 | 3 | 15 |
| % choice | – | 70.2 (7.6) | 87.7 (5.6) | 86.9 (5.2) | 89.2 (4.2) |
| % rating | – | 9.4 (8.9) | 55.4 (9.9) | 46.6 (10.8) | 53.7 (11.2) |
| % RT | – | 1.6 (2.3) | 12.0 (5.2) | 12.4 (5.6) | 12.0 (5.3) |

Note. n (parameters) = number of parameters per model; BIC = Bayesian Information Criterion (averaged over participants); n (best BIC) = number of participants, for which the respective model had the lowest BIC value; % choice / % rating / % RT = average percentage of trials, in which the peak of the choice probability function of the respective model matched the actual choice / number of sampled ratings / 100 ms bin (chance levels are: 50.0% / 16.7% / 2.5%). Note that all models have one parameter less than the models of the EEG study, since rating costs were constant across all trials making parameter α (see Equation 6) dispensable. Values in parentheses are standard deviations.


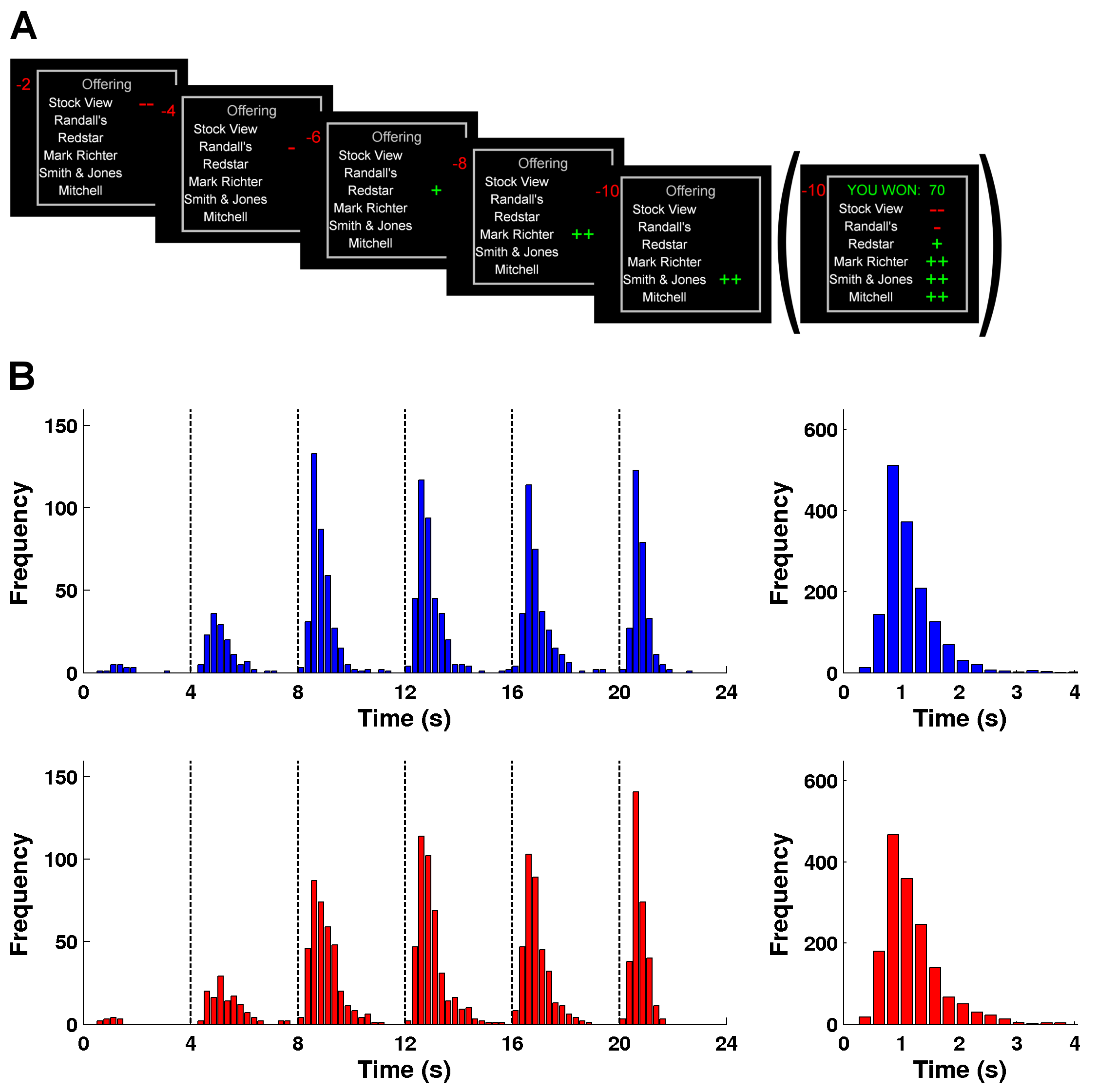


**Figure S1. Task and response time distributions of the fMRI study. (A)** Example trial of the paradigm in the fMRI study. Though the layout was more complicated, the task is very similar to the EEG study. The main differences are that there was only one cost condition and that ratings were presented longer (2 to 4 s). The feedback slide (in brackets) was only shown during training. **(B)** Reaction time histograms for buy (blue) and reject (red) decisions summed over all participants. Note that the length of a single trial was not up to 24 s but 17 s due to the variable presentation time.


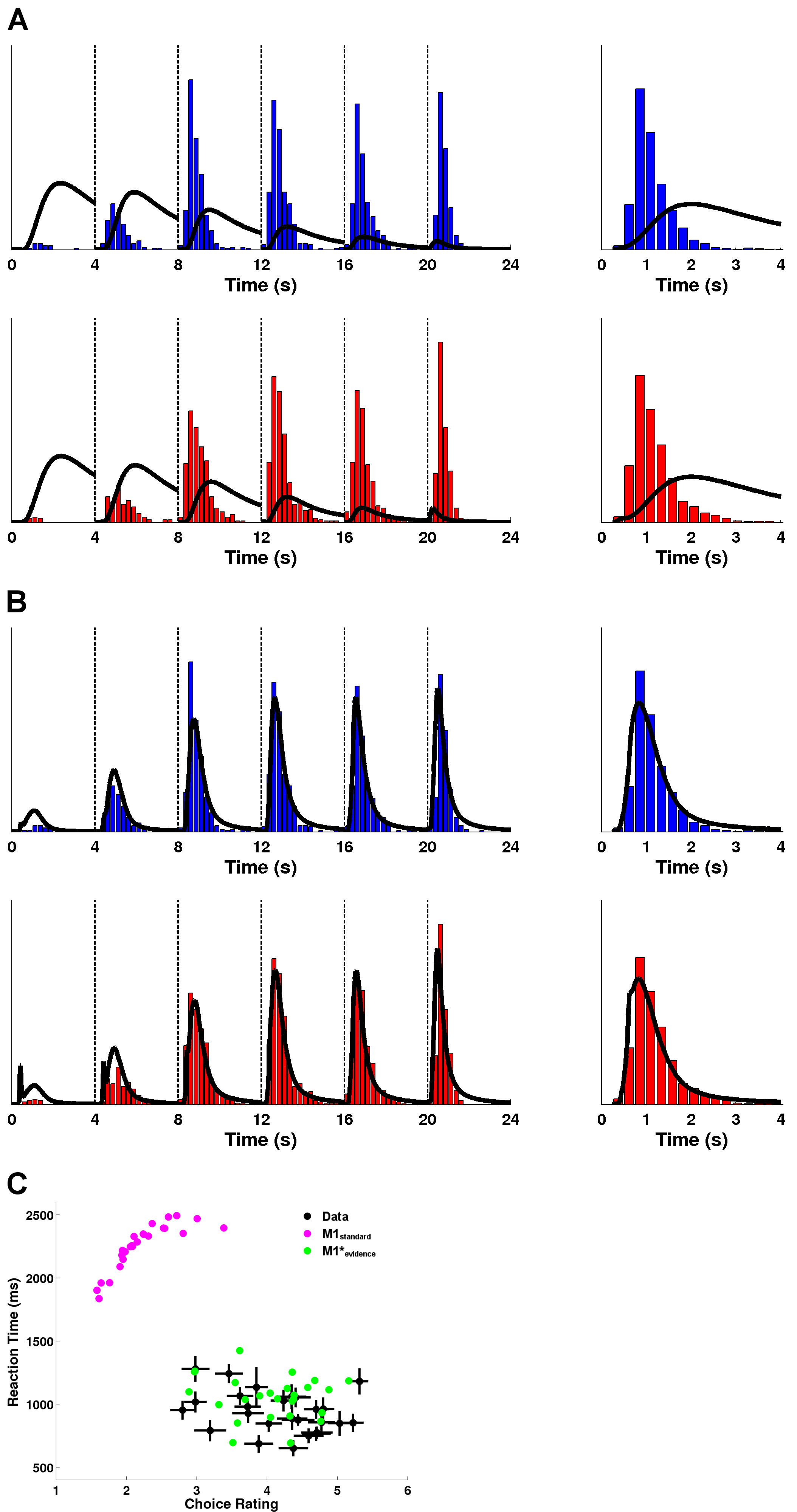


**Figure S2. Model comparison for the fMRI study.** **(A)** RT histograms of buy (blue) and reject (red) decisions as well as model predictions of the M1_standard_ model (when fitted to predict RTs). **(B)** Same as in **(A)** but with predictions of M1*_evidence_. **(C)** Average choice point in terms of rating number (x-axis) and mean RT (y-axis) per participant together with the respective predictions from the models M1_standard_ and M1*_evidence_.


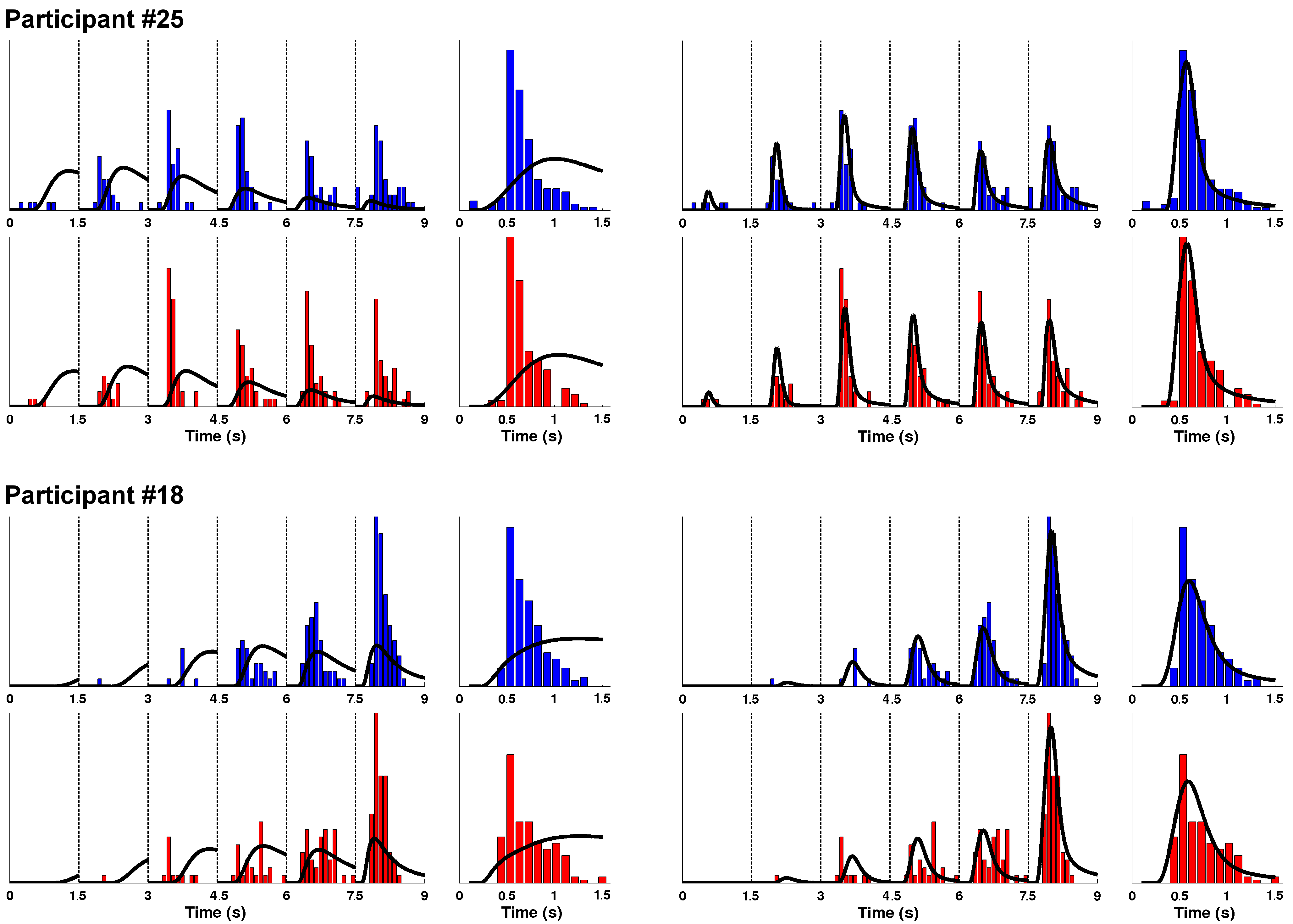


**Figure S3. Model fit for two individuals (EEG study).** Left panels refer to the model M1_standard_, right panels refer to M1*_evidence_. Participant #25 has a very narrow time window (within each rating), in which most of the responses are made. In contrast to M1*_evidence_, M1_standard_ cannot account for the steep decrease in response frequency after approximately 700 ms. Participant #18 sampled many ratings and mostly decided at the last rating. This makes it difficult for M1_standard_ to capture the RT distributions at earlier ratings.


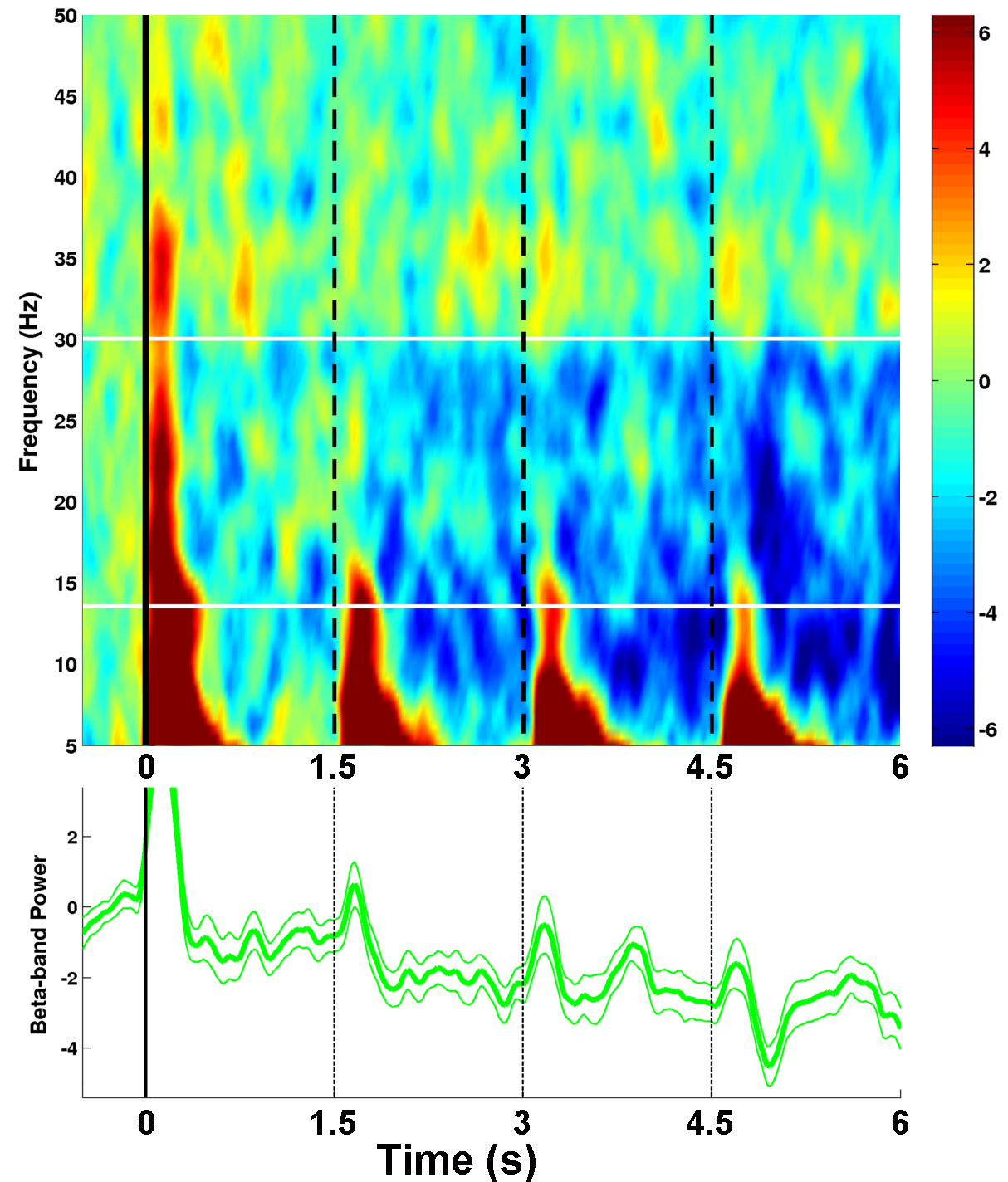


**Figure S4. Phase-locked time-frequency analysis.** Analogous to Figure 7, the upper panel shows the spectral power averaged over all scalp electrodes for the first four ratings, baseline corrected to the 500 ms before the first rating. The lower panel shows the average power in the beta-band. Whereas the general negative trend is also present in the phase-locked analysis, there is no evidence for an alternation between de- and increases at every rating (as seen in the non-phase-locked analysis). Indeed, no channel showed this pattern for beta (also not for alpha or gamma), neither at the threshold of *p* < .001 nor at *p* < .05.
